# Low-Stakes, Growth-Oriented Testing in Large-Enrollment General Chemistry 1: Formulation, Implementation, and Statistical Analysis

**DOI:** 10.1021/acs.jchemed.3c00993

**Published:** 2024-07-25

**Authors:** Tricia D. Shepherd, Sean Garrett-Roe

**Affiliations:** Department of Chemistry, University of Pittsburgh, Pittsburgh, Pennsylvania 15260, United States

**Keywords:** First-Year Undergraduate/General, Proficiency-Based Grading, Item Response Theory

## Abstract

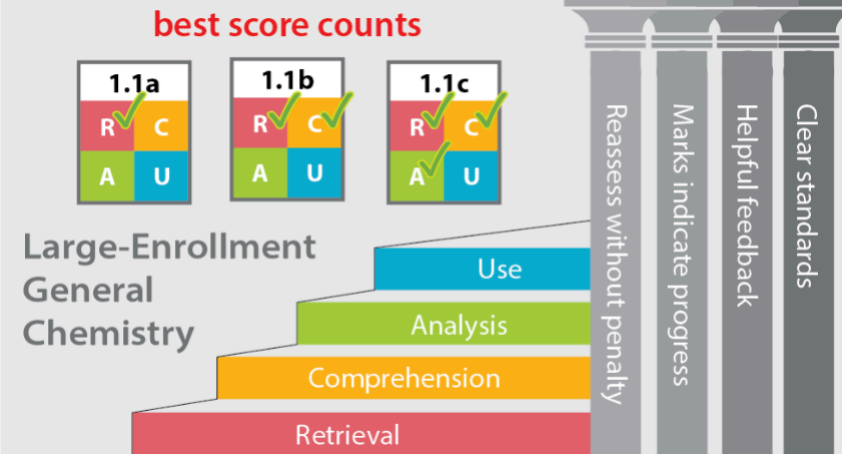

We formulate an alternative to high-stakes examinations that is designed to help students grow, and we describe its implementation in a large-enrollment General Chemistry 1 class. In our alternative grading approach, students complete weekly assessments. Each assessment has four items that are aligned to explicit learning objectives and a level in Marzano’s taxonomy, *retrieval*, *comprehension*, *analysis*, and *knowledge utilization*, which can be used by students and instructors to gauge the progression of student learning. Proficiency-based grading and multiple attempts reduce the stakes of the assessments. Unique assessments are generated through a computational infrastructure that draws question stems from an item bank and further randomizes quantities, elements, compounds, reactions, spectra, Lewis structures, orbitals, etc. in the questions. Nearly all assessment items require student-generated responses and cover a complete General Chemistry 1 curriculum. We interpret Marzano’s taxonomy in the General Chemistry context and outline the structure of the learning objectives, cognitive levels, assessment schedule, and grading scheme. Item response theory (Rasch analysis) validates the theoretical framework and indicates that assessment items are high quality. Students demonstrate improvement through assessment retakes, and they report that the system motivates them to study and learn.

## 1 Introduction

The General Chemistry curriculum, in all its aspects–curriculum, classroom pedagogy, laboratory, and assessments–has been the target of redesign efforts for decades.^1−7^*Learner-centered pedagogies* have been developed in the classroom^8,9^ e.g., Process Oriented Guided-Inquiry Learning (POGIL),^10,11^ Peer-led Guided Inquiry,^12^ Peer-Lead Team Learning (PLTL),^13^ Cooperative Learning,^14,15^ and flipped classes,^16^ and the laboratory,^17−20^ and they better support student learning than teacher-centered pedagogies.^9^*Learner-centered assessment*, on the other hand, is in its infancy, especially in the context of large-enrollment chemistry courses. Here, we demonstrate an assessment system for a large-enrollment first-term General Chemistry course that can both support and challenge students of all ability levels to grow as learners.

We are by no means alone or the first to embrace the new paradigm of learner-centered assessments.^21−25^ As Clark and Talbert^25^ explain, alternative grading systems have four pillars:

1. **Clearly defined standards.** “Student work is evaluated using clearly defined and context-appropriate content standards for what constitutes acceptable evidence of learning.”
2. **Helpful feedback.** “Students are given helpful, actionable feedback that the student can and should use to improve their learning.”
3. **Marks indicate progress.** “Student work doesn’t have to receive a mark, but if it does, the mark is a progress indicator toward meeting a standard and not an arbitrary number.”
4. **Reassessment without penalty.** “Students can reassess work without penalty using the feedback they receive until the standards are met or exceeded.”

Standards-based grading,^21,26−29^ specifications grading,^22−24,30^ and mastery-based testing^31,32^ follow these principles. Approaches like these have been demonstrated in large-enrollment chemistry lectures^26^ and laboratories.^28,29^

Inspired by Toledo and Dubas,^23^ our alternative assessment approach supports student progress toward higher-order thinking via following assessment structure:^23,24^

1. **Assessments are explicitly aligned to learning objectives.** Each class, homework, and assessment aligns to those objectives specifically.
2. **Assessments regularly test for low- and high-level cognition.** We write each assessment item to test thinking at a level of Marzano’s cognitive taxonomy,^23,24,33^ and each assessment presents students with one item at each of the four cognitive levels.
3. **Assessments are graded proficient or not-proficient with no partial credit.** Grading is focused on whether students demonstrate mastering the learning objective, and minor errors are allowed.
4. **Students have up to three attempts at questions aligned to each learning objective.** Because the grading is dichotomous (proficient or not proficient with no in-between), students have three chances to demonstrate their proficiency, and their best result is their score.

We aim to capture the spirit of Toledo and Dubas^23^ while operationalizing it for assessments in a large-enrollment course.

We demonstrate that our system encourages students to grow by demarcating a path to learn the discipline that leads up the levels of cognitive control. It supports the least prepared students because every assessment probes for foundational skills and understanding, proficiency at which is sufficient to pass the course. Simultaneously, it challenges the most able students because each assessment includes questions that elicit high-level critical thinking, scientific communication, and other scientific practices,^34^ proficiency at which is required to achieve the highest course grades.

To allow our system to be applied at scale, meaning hundreds of students in multiple parallel sections, we have developed an infrastructure to generate unique assessments aligned to the learning objectives. For each assigned learning objective, we select a random question stem, which is further randomized with custom quantities, compounds, reactions, Lewis dot structures, molecular orbitals, et cetera, covering the range of our General Chemistry 1 curriculum. A variety of different question types are used and almost all require student-generated responses. The computer infrastructure creates unique assessments for each group of students and a solution key with grading advice for the graders.

This infrastructure provides a unique stream of data back from our students, which allows us to characterize the overall assessment framework, to evaluate the individual assessment items, to observe student improvement, and to glean teaching insights. We wrote questions aligned with Marzano’s taxonomy, and we observe the predicted hierarchy in student response data, which validates our theoretical framework. Additionally, the robust numbers of student responses demonstrate that the assessment items are high quality. Finally, these data show first insights into how the system allows students to improve.

In this paper, we report the structure of our assessment system, show that our items both align with the learning taxonomy and are high quality, and demonstrate both how our system supports student learning and that students at all performance levels can improve.

## 2 The Structure of the Assessment System and Its Implementation

### 2.1 Instructional Setting

The assessment system was implemented at a large, state-related research university in the northern United States. Two sections of first term General Chemistry 1 were transformed in 2021 and one section in 2022. All data are from the 2022 iteration (∼250 students). The students are 74% women, 22% men, and <5% nonbinary or did not report gender; 17% of students are classified by the university as underrepresented minorities; 12% are first generation college students; 12% are Pell eligible. Students have not declared their majors, but only a few percent become chemistry majors on average; most intend to pursue a career in the health sciences. Students attend two 75 min in-person classes conducted using POGIL activities Moog et al.^35^ and custom activities, and learning teams of ∼4 are supported by a team of 11 cofacilitators (10 undergraduate and 1 graduate teaching assistants). The in-class structure is the same as described in ref (36)., which showed short- and long-term benefits for our student population, except now all in-class experiences include POGIL. In addition, students meet once per week in groups of 24 for a 1 h recitation led by the graduate teaching assistant followed by a 3 h laboratory experience, which was not transformed. The course follows a traditional reactions first (not atoms first) curriculum using the OpenStax “Chemistry 2e”^37^ text. In 2022, we used the ALEKS adaptive learning system^38^ with ALEKS learning objectives selected to best align with the course objectives. We classify most ALEKS items at the *retrieval* and *comprehension* levels. Only a few learning tasks in ALEKS meet the *analysis* level, and none fit the criteria for *knowledge utilization*. The learning management system was Canvas, in-class questions were delivered through TopHat,^39^ and all grading was completed in Gradescope.^40^

The assessments compose half of the student’s course grade. The other half of the grade is based on the laboratory (20%), homework (ALEKS, 15%), in-class participation (TopHat, 10%), and American Chemical Society (ACS) standardized exam (5%). The details of calculating grades are given in Supporting Information (Section S4).

### 2.2 Organization of Learning Objectives in Marzano’s Taxonomy

Our assessment approach employs a learning taxonomy with a hierarchical structure. The four cognitive levels – *retrieval*, *comprehension*, *analysis*, and *knowledge utilization* – are based on the mental operations or conscious processing applied to knowledge rather than an inherent degree of difficulty or complexity in the question itself.

Briefly, the lowest level, *retrieval*, focuses on the recognition or recall of information or executing procedures without necessarily understanding why the procedure works. The next level, *comprehension*, requires a deeper understanding of the basic structure of the knowledge. Students should be able to identify critical components of the information and be able to encode and decode it in nonlinguistic or abstract form. *Analysis* tasks require the learner to examine knowledge in fine detail and reorganize the information in a way that generates new conclusions. Finally, the highest cognitive level, *knowledge utilization*, requires students to apply or use knowledge to make decisions or solve problems. The key distinction from analysis is a shift in focus from the knowledge itself to a specific situation in which the knowledge can be employed. Our interpretation of Marzano’s taxonomy in the Chemistry context is provided in the Supporting Information (Section S1).

### 2.3 Student Learning Objectives

**Figure 1:**
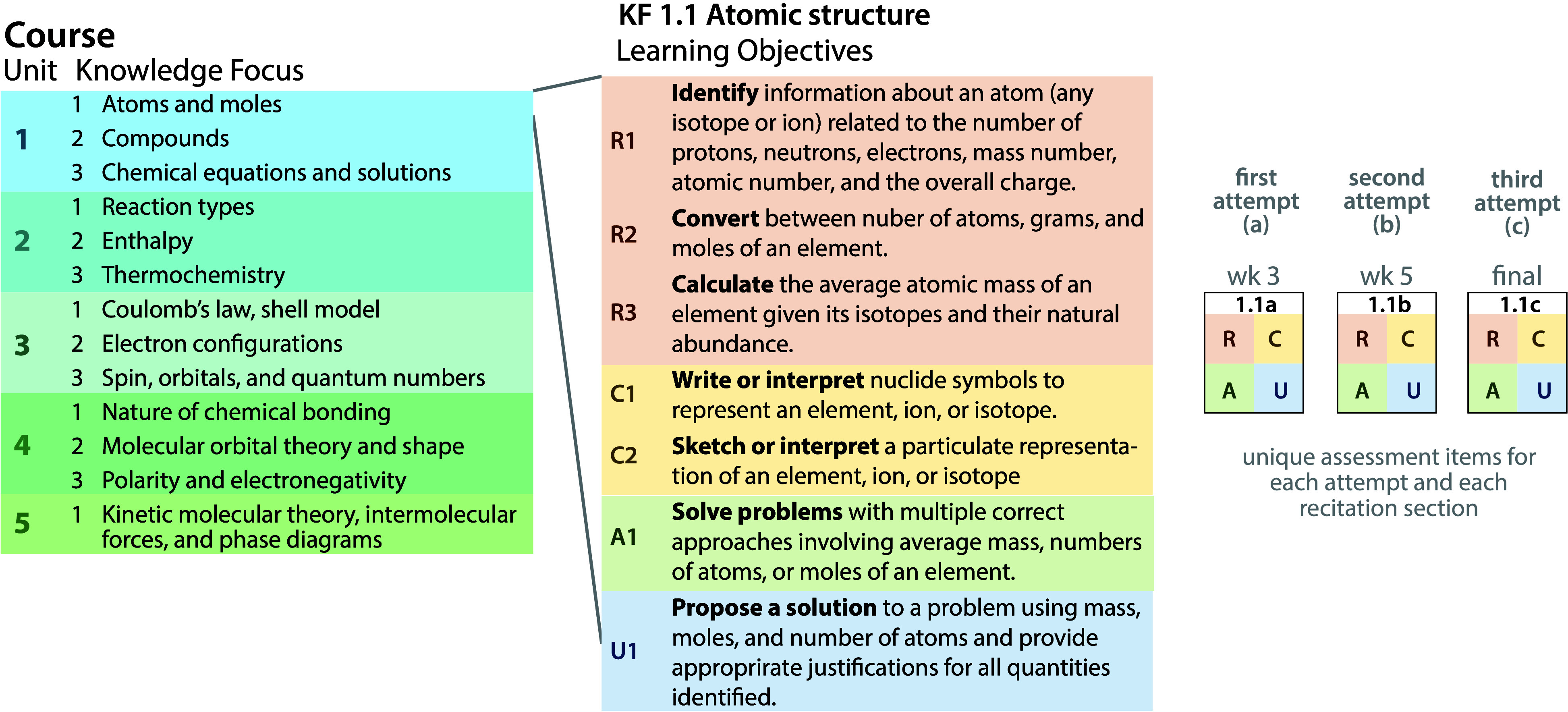
Division of the course into Units, Knowledge Focuses, Learning Objectives, and their aligned assessments. (left) The five thematic units contain 1–3 Knowledge Focuses. (center) Each Knowledge Focus is a set of learning objectives expressed at the levels of Marzano’s taxonomy – *retrieval* (R), *comprehension* (C), *analysis* (A), and *knowledge utilization* (U). (right) Assessments draw one question at each level from the set of objectives in the Knowledge Focus, and students have three attempts in the term.

We divided the General Chemistry 1 curriculum into five units, each roughly 3 weeks long (Figure 1, left). Each unit (except the last) is divided into three Knowledge Focuses^23^ each with a set of learning objectives to which an assessment aligns (Pillar 1: Clearly defined standards). The set of interconnected learning objectives is a Knowledge Focus,^23^ and there are 13 in total. Each Knowledge Focus has several objectives, at minimum one at each of the four levels of Marzano’s taxonomy (Figure 1, center). The course has 81 learning objectives (Supporting Information, Section S2). Assessments are built with four items, one at each of Marzano’s levels (Figure 1, right).

### 2.4 Question Bank Design and Implementation

The sheer number of students in a the large-enrollment course makes delivering low-stakes assessments challenging. In our case, assessments are delivered to students in-person during the recitation period. Each week, students divide into 11 recitations per course section, which are scheduled across the week. To minimize academic integrity violations, each recitation receives a unique assessment. Assuming each assessment has four items, there is one assessment for each of 13 Knowledge Focuses, and students have three attempts at each assessment, this course would require 1716 unique questions–a daunting task for any instructor.

To create questions on this scale, we developed a computer infrastructure to generate the assessments. We wrote 300 question stems (96 *retrieval*, 70 *comprehension*, 72 *analysis*, and 62 *knowledge utilization*) to cover the five units, 13 Knowledge Focuses, and 81 learning objectives of the course. Nearly all questions require a student-generated response in the form of calculations, drawings, and symbols. Using the codes proposed by Ralph et al.,^41^ 43% of items require “math”, 40% require “mechanistic reasoning”, 7% require both, and 24% require neither. A detailed characterization of question types and coding is provided in the Supporting Information (Section S1.3). Each question stem is a template written in LaTeX, and the template is filled in programmatically with custom Python code using pythontex.^42,43^ The LaTeX templates use many freely available packages to correctly typeset chemical formulas, equations, and structures (notably mhchem,^44^chemfig,^45^chemmacros,^46^ and modiagram(47)). Packages such as siunitx provide support for scientific units, and tikz provides general drawing functionality. The python code draws heavily on the open-source packages for scientific computation (SciPy and NumPy) and chemistry specific python libraries including ChemPy,^48^ pymatgen,^49^ and periodictable.^50^

The questions cover the range of our General Chemistry 1 curriculum. In addition to randomizing quantities, the engine selects elements, compounds, and reactions. Lewis structures, including resonance structures and formal charges, can be generated. It can choose systems of equations for Hess’s Law problems, calculate thermodynamic quantities from thermochemistry tables. The system can draw shell models and photoelectron spectra of atoms, atomic orbitals, hybrid orbitals, molecular orbitals, and molecular geometries. It serves our needs for all General Chemistry 1 content.

Even subtle changes in questions can substantially alter their difficulty.^51^ While we randomize quantities, elements, reactions, etc., we used pilot data to remove obvious outliers. The analysis below represents the difficulty of these items averaged over all their instances.

The infrastructure compiles the student assessment and an instructor solution with grading suggestions. The questions given as practice problems in the recitation are excluded from the assessments, and students are assigned a specific *analysis* and *knowledge utilization* question only once.

### 2.5 Learning, Assessment, and Reassessment

**Figure 2:**
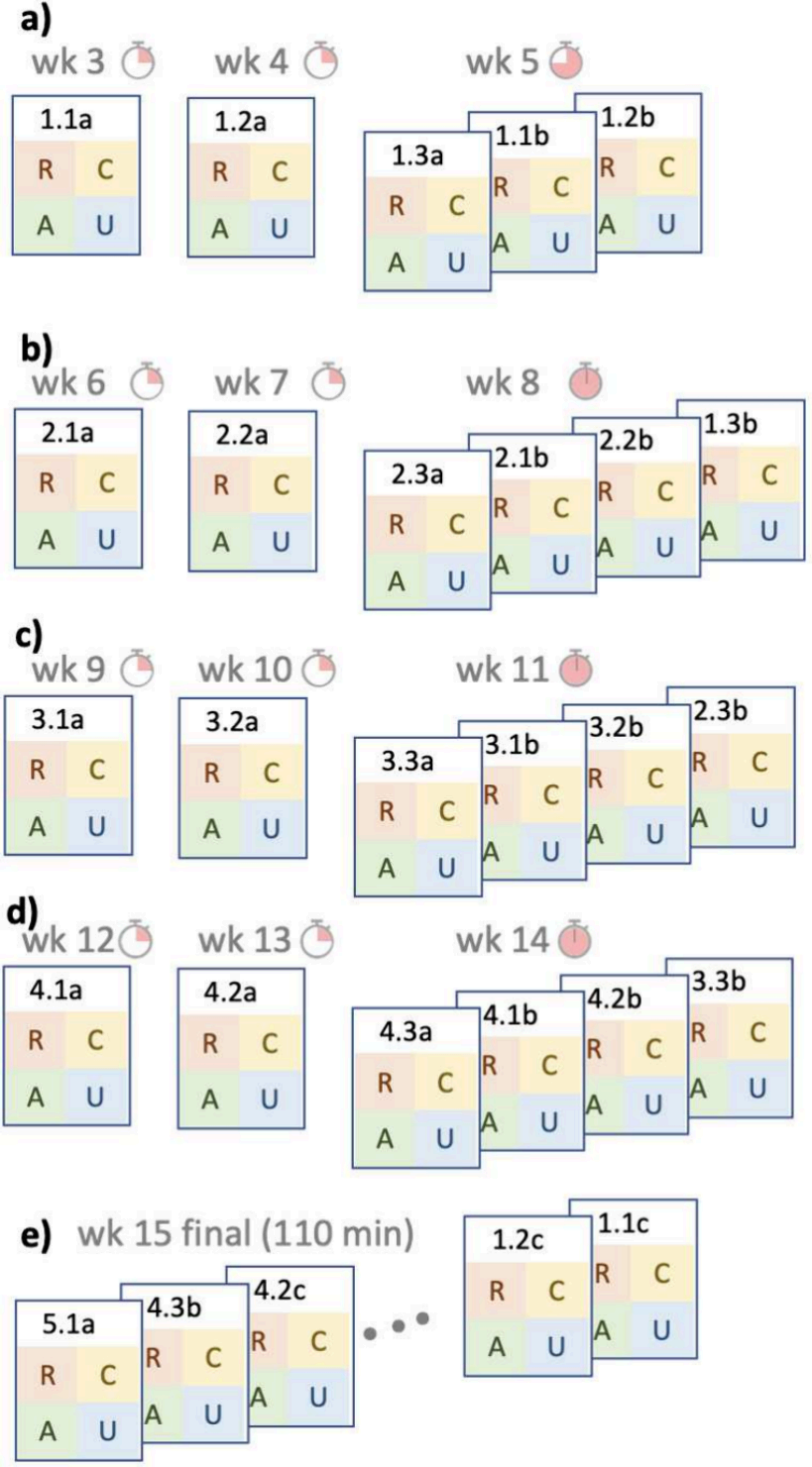
Schedule of assessments. a) Students take the first attempt at Knowledge Focuses 1.1, 1.2, and 1.3 in weeks 3, 4, and 5. In week 5, students additionally may attempt retakes of 1.1 and 1.2. b) In week 8, students complete the first attempt at 2.3 and second attempts at 2.1, 2.2, and 1.3 (from the previous unit). d,e) The pattern repeats again for units 3 and 4. e) During the final exam period, all assessments are available to students.

The in-class activities, homework, recitation practice, and assessments occur in a deliberate sequence to support student learning. Ideas are introduced in class with POGIL activities. That week, students complete homework (ALEKS) to practice the skills developed in class. In the following week, students attend recitation and work problems aligned with the learning objectives in their learning teams supported by the graduate teaching assistant. In the subsequent recitation, students have 15 min during the in-person recitation to complete the four questions (proctored by the graduate teaching assistant). At the end of the unit, students have a second assessment with randomized questions aligned to the learning objectives in the Knowledge Focus (Figure 2). This pattern repeats through the term–learning new content in class, practicing through the online homework system, refining understanding in recitation, and demonstrating learning on the assessments–for each Knowledge Focus. The final attempt is delivered during the final exam period, during which students are free to complete as many assessments as time allows (110 min).

Every third week, students complete one new assessment and up to three retakes (Figure 2). As a consequence, on these weeks (5, 8, 11, and 14) the assessments use most or all of the recitation time. Students are still provided a recitation activity and also worked solutions to help study for the next week’s assessment. We also note that students have only two attempts at Knowledge Focus 4.3 and one attempt at 5.1.

### 2.6 Scoring Rubric and Feedback

Students receive structured feedback through a rubric and additional feedback from the graduate teaching assistant (Pillar 2: Helpful feedback). Each of the four student answers is marked on a five-point rubric: (1) Correct (solution provided); (2) Proficient with minor error; (3) Some progress toward learning objective, not proficient; (4) Little evidence of progress toward learning objective, not proficient; (5) No response. The first two items of the rubric are awarded full credit, and the following three are awarded no credit. The rubric item for correct responses also displays to all students the correct answer and a worked solution, if applicable.

**Figure 3:**
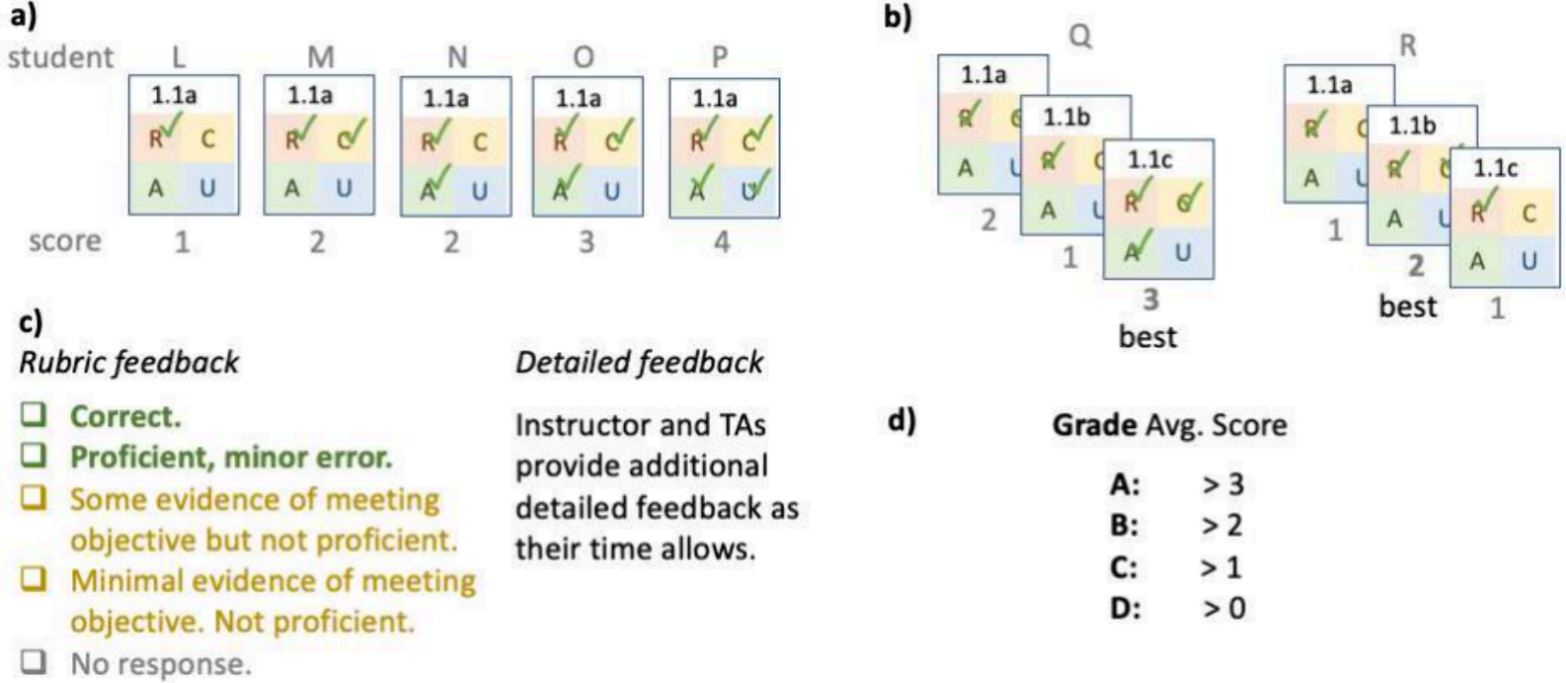
Assessment score, feedback, and grade. a) Each student’s assessment is scored 0–4 by the number of proficiencies demonstrated. b) The best score of all attempts, regardless of order, is the only score that contributes to the student’s grade for the course. c) Each item answer is marked on a rubric with 5 levels. d) The course grade cutoffs are chosen to map average assessment scores to particular grades. For example, averaged over all assessments, students scoring >3 earn an A, > 2 earn a B, > 1 earn a C.

The system encourages students to try again (Pillar 4: Reassessment without penalty). Each assessment is scored by the number of proficiencies students demonstrate (Figure 3), and only the best score contributes to the grade.

The mark for each assessment indicates a student’s progress toward high-order thinking in that Knowledge Focus (Pillar 3: Marks indicate progress). When a student repeats an assessment, they are presented with new *retrieval*, *comprehension*, *analysis*, and *knowledge utilization* questions, and they must again demonstrate proficiency at all the levels. Students cannot “cherry pick” questions, i.e., only answer questions they deem “easy.” A student who scored 2 on a first attempt, (Figure 3b, Student Q) will need to show that they are still proficient at *retrieval* and *comprehension* and also have new proficiency at higher levels to increase their score on that Knowledge Focus. If a student does not show their proficiency again, they are not penalized (only the best score counts toward their grade), but neither can they improve their grade. The system encourages students to retain their knowledge because students have to repeat their mastery of lower-level objectives while working toward higher-level objectives.

## 3 Results

### 3.1 Taxonomy Is Hierarchical

**Figure 4:**
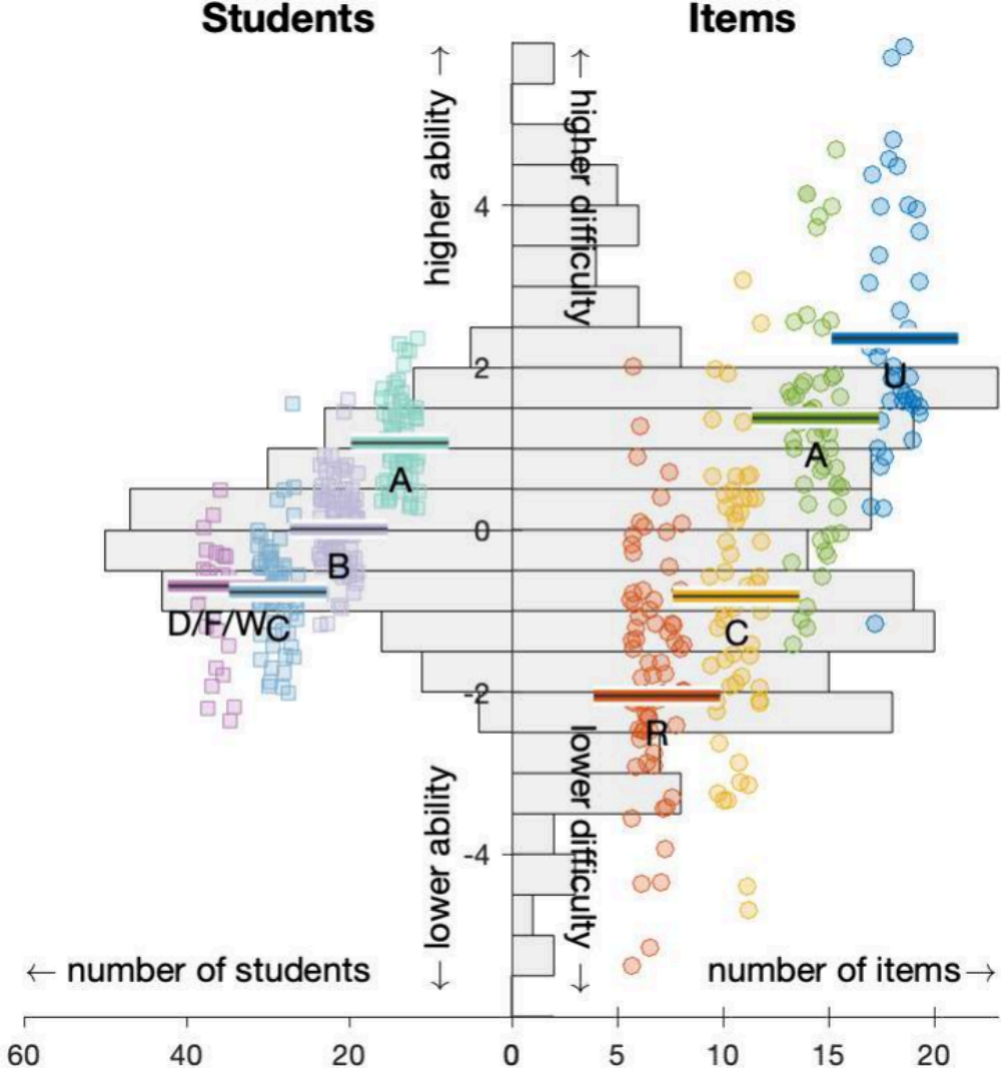
A Wright Map from the Rasch analysis with a histogram of (left) student ability overlaid with individual student course scores separated by grade – D/F/W (pink), C (cyan), B (lilac), A (teal) – and (right) item difficulties overlaid with individual items colored by categorization in Marzano’s taxonomy–*retrieval* (R, red), *comprehension* (C, yellow), *analysis* (A, green), *knowledge utilization* (U, blue).

Student and item performance were assessed with item response theory^51−53^ based on students’ first attempts at the assessments (Figure 4). Item response theory evaluates both student ability and item difficulty (Supporting Information, Section S5). Item response theory presents ability as a latent trait at a snapshot in time. As practitioners, we conceive of student ability as a quantity that grows through student effort. Nevertheless, we adopt the term ability to match the technical language of the field.

In that context, item response theory presents both student ability and item difficulty on the same axis. On this unified scale, a student will have a 50% chance of correctly answering an item when that student’s ability estimate equals the item’s difficulty. Students with ability estimates higher than the item difficulty are more likely to answer correctly than not, and, conversely, are unlikely to correctly answer questions of difficulty greater than their estimated ability.

Student ability estimates are peaked near 0 on the logit scale, and 52% the population falls within the range −1 to 1 (Figure 4, left histogram). The ability estimates of 241 students are plotted as squares, grouped and colored by their final course score.

The students with similar final course scores tend to have similar ability scores. Students with B’s have a median proficiency in the center of the range, 0.01 (indicated by the horizontal line of the group’s color), and the medians for A’s and C’s are one unit above or below that (median A 1.07, median C – 0.77, median D/F/W – 0.69). Students in each grade group are scattered one unit around the median proficiency level for that group. The score clusters for students earning C’s and D/F/W’s are similar. Students in the D/F/W group typically performed poorly in other course components, especially the homework and laboratory.

The assessment items follow the predicted hierarchy (Figure 4, right). The assessment items cover a wide range of difficulties (right histogram). The estimated difficulties of 244 items in the bank used on first attempts are also shown as circles, grouped and colored by the taxonomy level. The median difficulties of each level increase in order *retrieval* (−2.05), *comprehension* (−0.82), *analysis* (1.38), and *knowledge utilization* (2.36). This stepwise progression is strong evidence that our coding system meaningfully categorizes items with different levels of cognitive complexity. The same trends are obtained when all attempts are included (Supporting Information, Figure S1).

These ability and difficulty estimates immediately provide insight into what students at each grade level are likely to demonstrate on an assessment. The Rasch model (Supporting Information, Equation S1) gives a predicted probability that a student with a certain ability level will be able to successfully answer a question at a given difficulty level. Based on the median ability and difficulty for each group, the model predicts that students earning C’s have a roughly 78% chance of demonstrating proficiency at a *retrieval* question, 51% at *comprehension*, and only 10% and 4% at the higher cognitive level *analysis* and *knowledge utilization* questions. At the other end of the spectrum, students earning A’s have a predicted 96% proficient rate at *retrieval*, 87% at *comprehension*, and 42% at *analysis*, and 22% at *knowledge utilization* on the first attempt at that learning objective.

**Figure 5:**
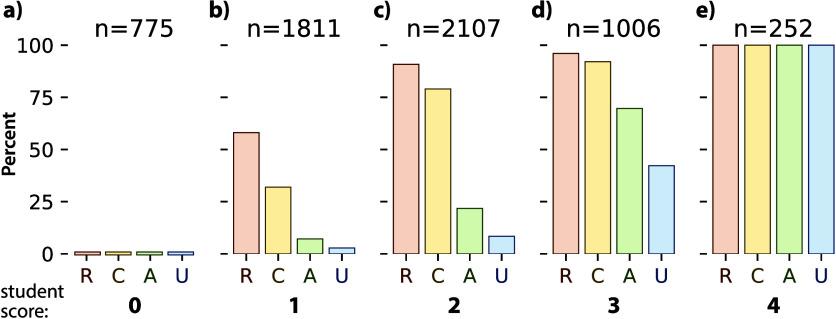
Percent of students demonstrating proficiency at *retrieval* (R), *comprehension* (C), *analysis* (A), and *knowledge utilization* (U) level by score: a) score = 0, b) score = 1, c) score = 2, d) score = 3, e) score = 4.

The observed student scores confirm these statistical predictions (Figure 5). Students who demonstrate a single proficiency at a learning objective (score of 1) overwhelmingly are proficient at the *retrieval* (58%) and *comprehension* (32%) levels and rarely at the *analysis* and *knowledge utilization* levels (Figure 5b). Students who demonstrate three proficiencies (score of 3) have a high likelihood to master *retrieval* and *comprehension* questions (>90%) and are more likely to show proficiency at *analysis* than *knowledge utilization* (Figure 5d). This performance data supports our interpretation of *retrieval* and *comprehension* as relatively lower level thinking skills and *analysis* and *knowledge utilization* requiring increasing levels of cognitive control.

The point of this statistical characterization is to validate the grouping of questions into the hierarchy proposed by Marzano. We do not consider student “ability” to be a fixed, latent trait, though the technical terminology of item response theory might imply that. Section 3.2 will, indeed, show the improvement that students achieve in our system.

**Figure 6:**
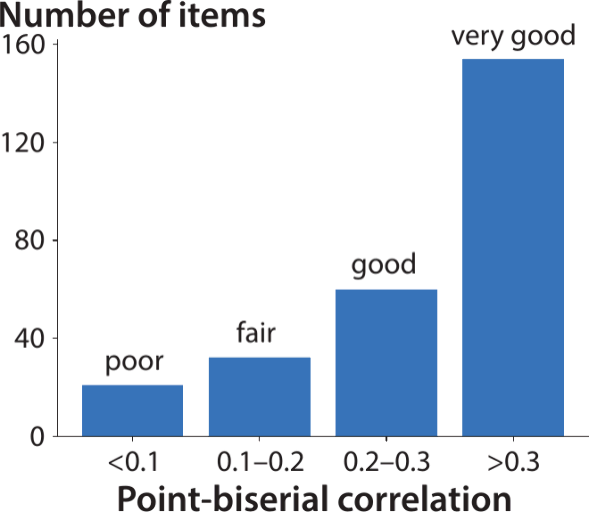
Histogram of point-biserial correlation values for all questions in the item bank. Partitioning is aligned with that of Sorenson and Hanson.^52^

The assessment items in the bank are overwhelmingly high quality (Figure 6). Point-biserial correlation analysis^52^ shows that 80% of items in the question bank are good or very good, and only 8% are poor, similar to a question bank that was iteratively improved based on item response theory.^52^ Examination of the questions rated as poor did not reveal any consistent reason for the low-rating. We applied the guidelines for high-quality multiple choice items^54^ and did not observe obvious flaws in stem construction (confusing structure, use of negatives, etc.); of course, the questions could be flawed in ways we do not yet perceive. Remaining explanations include poor statistics (low response numbers, very high or low success rates), unreliable grading decisions, and common misconceptions that can be addressed in teaching interventions.

### 3.2 Students Improve

Having validated the organization of questions in the taxonomy with Rasch analysis, we analyze the ability of students to improve when they retake assessments.

**Figure 7:**
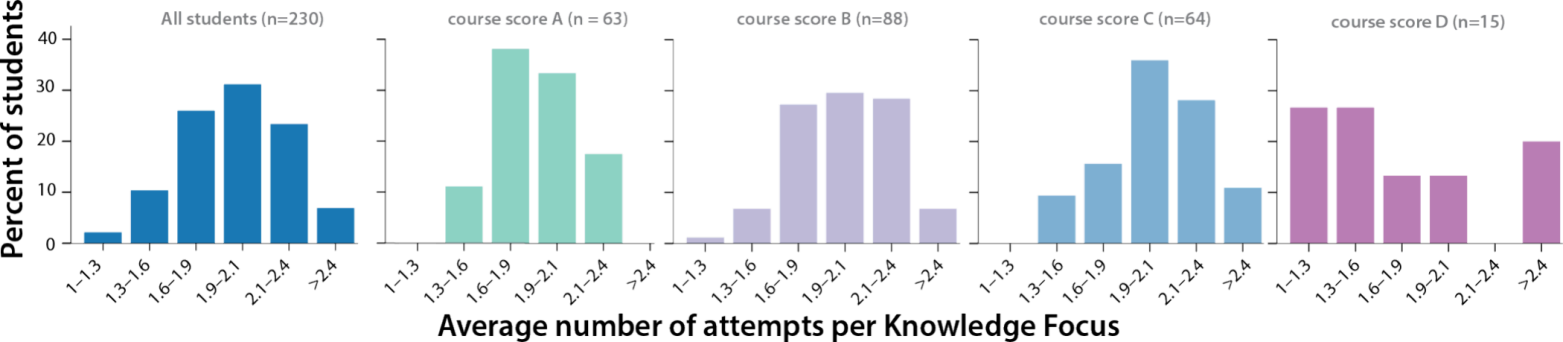
Histograms depicting the number of attempts per Knowledge Focus. All students (left, dark blue). By final course grade: A (center left, teal), B (center, lilac), C (center right, blue), and D (right, pink).

Most students take each assessment about twice (Figure 7). Considering all students, the most likely number of attempts per Knowledge Focus is peaked around two attempts, and a student’s score in the course has only a mild effect on the distribution. The average number of attempts for students earning an A is slightly less than 2 attempts, students earning a C have an average slightly above 2 attempts, and the distribution for students earning B is evenly distributed between 1.6 and 2.4 attempts. The broadest distribution is for the students who earned a D, though these numbers are small. Almost all students try again to improve their score.

**Figure 8:**
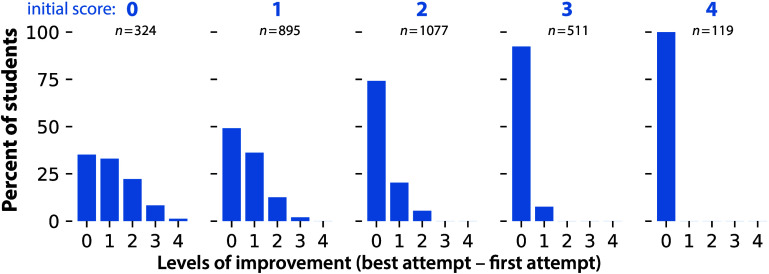
Histograms of levels of improvement (the student’s best score minus the first attempt score (0 to 4 levels)), by initial score: 0 (left), 1 (middle left), 2 (middle), 3 (middle right), 4 (right).

Students with low initial scores show the greatest improvement (Figure 8). Of students who score 0 on their first attempt (Figure 8, left), 65% of students improve, usually by 1 or 2 levels (Figure 8, left); improvements of 3 levels are uncommon and 4 levels are rare. Fully 50% of students who score a 1 (middle left) on their first attempt improve, and 25% of students scoring 2 on their first attempt (middle) improve. Only 8% of students scoring a 3 on their first attempt (middle right) improve by 1 level, reaching the maximum score of 4. Students who initially score a 4 (right) cannot improve because their first attempt is already the maximum. We attribute the low numbers of students who initially score a 3 and then improve to a 4 to both the difficulty of getting a 4 and also to student satisfaction with a score of 3. This breakdown shows that our system most encourages mobility for the (initially) lowest performing students.

The first attempt score has a low positive correlation with overall course score (Pearson correlation coefficient^55^ 0.47), and students across a wide range of course scores improve (Supporting Information, Figure S2).

**Figure 9:**
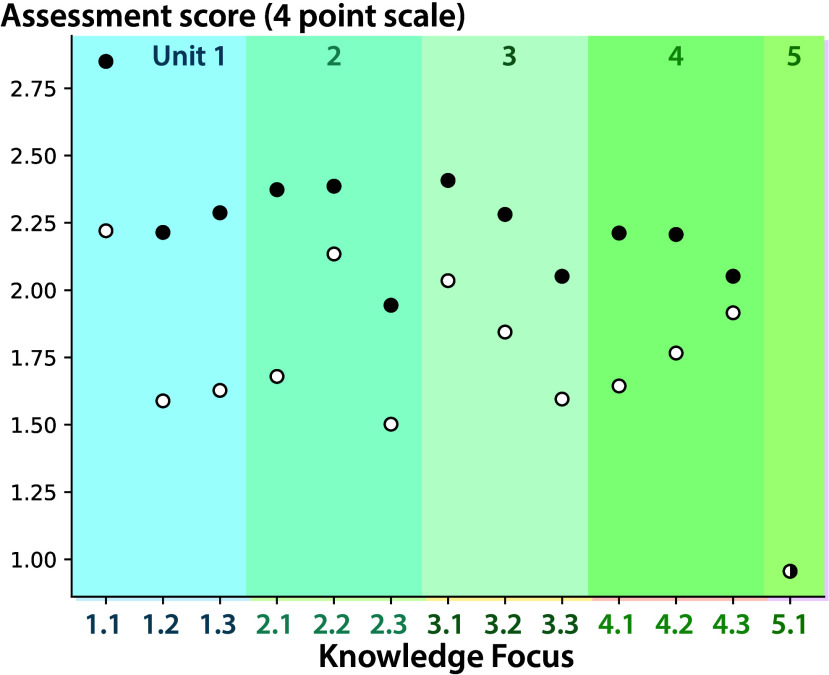
Average assessment scores for all Knowledge Focuses. Student first attempts (open circles) and best attempts (filled circles).

Improvement occurs across the term. On their first attempts, students score ∼1.7 on each Knowledge Focus on average (Figure 9, open circles). Knowledge Focus 1.1 (Atoms and moles) has the highest score for first attempts (2.2), likely because many students are familiar with these concepts from their high school chemistry courses. Unit 5 (Intermolecular Forces), at the end of the term, has the lowest first attempt scores (0.95). The assessment for this Knowledge Focus is completed during the final exam period and the high-stakes assessment environment may increase stress and reduce scores. Knowledge Focus 2.3 (Thermochemistry) has the next lowest first attempt scores (1.5) because many students struggle with calorimetry.

Students improve in all Knowledge Focuses (Figure 9, filled circles) by an average of ∼0.5 points across all assessments. (Students have only one attempt at Knowledge Focus 5.1, so the first attempt is also the best attempt.) The largest gains occur in the earliest units of the course. On Knowledge Focus 1.1 (Atoms and moles), students’ scores improve to >2.75, the highest average score. On Knowledge Focus 2.1 (Solution Reactions), students improve by ∼0.7 points, the largest average improvement.

Finally, the observed grades match the design targets. The average assessment scores of students earning passing grades are slightly above the *analysis*, *comprehension*, and *retrieval* thresholds, as targeted in the design (A: 3.10, B: 2.19, C: 1.55, and D: 1.32). Students earning grade D perform similarly to students earning grade C. The other course components – (in order of decreasing importance) lab, homework, class participation, and the ACS exam–become grade determining.

## 4 Discussion

### 4.1 Subjective Reflection on Teaching, Learning, and Grading

Students report liking the assessment system. In the end of term survey question, “What did you like best about how the course was taught?”, 26% of students responded that the assessments and retakes were what they liked best. Many students expressed that they wish other classes had retakes, they felt less stress, and the structure encouraged them to learn and do better. A small minority of students (6%) expressed that the all-or-none proficiency-based grading left them frustrated because their scores, with no partial credit, did not represent what they had learned.

Other reports of alternative grading systems have documented similar student perception in other chemistry courses. Link et al. described the benefits of specifications-based grading in pilot^28^ and large-enrollment^29^ organic chemistry laboratory courses from the perspectives of students and teaching assistants. Students perceived similar benefits, and a minority of students expressed similar reservations. Hunter et al.^56^ report that specifications-based grading and mastery-based grading in Analytical class and laboratory both help students move from memorization to a deeper demonstration of their knowledge. A difference is that students in our system have only a finite number of attempts, which may side-step feeling compelled to redo many assignments. Ahlberg^57^ similarly noted, “Many students indicated how these helped them to learn, helped hold them accountable, but without the pressure of a score,” in a specifications-based grading implementation of organic chemistry.

We appreciate the assessment system because it provides an accurate language to talk with students about how to improve and grow. Conversations with students can be framed in terms of the level of proficiency they have demonstrated and what the next steps in the hierarchy are, and this is all in the context of the specific learning objectives for that Knowledge Focus. With students who are not yet proficient at *retrieval* and *comprehension*, we can guide them toward the basic facts and understanding of the unit. For students who are moving toward proficiency at *analysis* and *knowledge utilization*, we can discuss the higher-level problem-solving skills and what it means to justify an answer clearly and concisely. These conversations feel efficient, productive, and personalized.

Creating and implementing this system has caused us to reflect on the meaning of grades in General Chemistry. In our traditional assessments, a student’s grade indicated their ability to answer many questions of middling difficulty quickly and correctly. We find the new grading system much more satisfactory. *Retrieval* and *comprehension* questions can be answered correctly at high rates, while *analysis* and *knowledge utilization* questions remain challenging even for very able students (Figure 4). Students earning a C, passing the course, can reliably answer questions at the level of basic facts and algorithmic calculations for the bulk of the course material and can explain the concepts (*Comprehension*) for at least some topics. Students earning a B can do those tasks reliably and can sometimes engage in complex problem-solving (*Analysis*). Students earning an A can complete those tasks reliably and also are challenged to use their knowledge creatively in a variety of scientific practices^34^ in new contexts (*Knowledge Utilization*). Our system allows us to communicate to our students a path of growth through these levels.

### 4.2 Potential for Research

The rich student performance data from our system could impact future educational research in General Chemistry. The assessment item infrastructure makes it feasible to track student response rates at the levels of item, learning objective, and Knowledge Focus. These can be monitored as a function of interventions in the classroom, student demographics, and student surveys. Connecting information about students with the wealth of student responses will change what one can measure from crude data, like how a factor affects a student’s final grade, to granular metrics, like how a factor affects a student’s ability to improve in certain areas or master certain cognitive levels.

Our system has potential to impact research on assessments in chemistry. Because we differentiate between the cognitive level (*retrieval* versus *comprehension*) rather than the format (symbolic versus particulate), we may be able to offer clarifications on the impact of representations on student performance and equity.^58^

The potential for mindset research is particularly clear. Where general mindset research relies on students’ self-reported attitudes and total course score, we can provide data on students’ measured growth on many assessments. “Entity theories” (fixed mindset)^59,60^ would predict that students of different natural abilities would always score at their level. Able students would score high on all their attempts, and less able students would score poorly on all their attempts. “Incremental theories” (growth mindset),^59,60^ however, would predict that all students, regardless of their starting ability, could demonstrate improvement. We observe both–many students improve, but some more than others. Our assessment system provides a potential link between student self-report of their mindset, mindset interventions,^61^ and how this mindset translates into measurable learning and growth. Using newly validated mindset instruments^59,60,62^ and our assessment system together may prove exceptionally fruitful.

Stress beyond a certain point is harmful for learning. Mindfulness interventions have been developed to mitigate stress in the chemistry class.^63^ Our approach is a potentially useful complement, in which stress is reduced by lowering the stakes of the assessments. Lewis^64^ showed evidence that specifications-based grading in mathematics courses can indeed reduce stress, though no change in growth mindset was detected.

The platform we have developed will also provide a robust system to test if low-stakes assessments can decrease performance gaps and increase equity in our classrooms. High-stakes assessments harm students,^25,65^ but we had no better assessments to replace them with in our large-enrollment courses. The system described here naturally provides assessment items that call on multiple kinds of cognitive ability (not just *retrieval* (execution) tasks), which Shah et al.^66^ highlight as an important ingredient for improving equity. It also provides formative feedback, allows mistakes, and is an “intrusive” teaching practice (in the positive sense), which are qualities emphasized by White et al.^67^ for increasing equity. Our approach is a “highly-structured” system, which itself can increase equity.^7^ Ralph et al.^41^ have discussed the impact that different question styles can have on the performance of students from underrepresented minority groups. They recommend that General Chemistry assessments increase the fraction of questions that elicit mechanistic reasoning. Our assessment system presents students with “mechanistic reasoning” tasks above the rate of the “Curriculum Reform” of Ralph et al.^41^ and “math” tasks at a similar rate (Supporting Information, Table S3), also suggesting potential benefits to underrepresented students. Now, we can test if a low-stakes system can, indeed, help us progress toward our goals for equity in the classroom, which has the promise to raise underrepresented students into a “hyperpersistent state”.^68^

## 5 Conclusion

We have demonstrated an alternative assessment system for large-enrollment General Chemistry 1. The approach uses low-stakes assessments (proficiency-based grading and multiple attempts at assessments), and it operates at scale. The assessments are aligned to learning objectives expressed at four levels of cognitive complexity, *retrieval*, *comprehension*, *analysis*, and *knowledge utilization*. Assessments are created for each Knowledge Focus by a computational infrastructure that draws a random question template at each of the four levels from an item bank and further randomizes the questions with quantities, compounds, reactions, and orbitals. Item response theory (Rasch analysis) validates the hierarchy in Marzano’s taxonomy and demonstrates the high quality of the assessment items. Students improve through the retake process, demonstrating that the system encourages learning and growth.

Though we use a particular active learning pedagogy, POGIL, in the classroom, this assessment approach is potentially applicable for any classroom technique. It should provide a benefit for any instructional style and a wide variety of STEM disciplines. Students can learn and improve on assessment retakes regardless of the style of their classroom. It is an open question whether student-centered or traditional instructional styles will benefit most from our alternative grading approach.

Our system is a blueprint for how to deploy alternative grading in large-enrollment courses. We show that alternative assessments can be both flexible and also challenging. Our ultimate aim is to empower all students to take charge of their learning, encourage their growth as scholars, and welcome them into the academic community of science. While the impact could be greatest in large-enrollment courses, the framework we demonstrate could be adopted in classrooms of any size.

## Supporting Information Available

Interpretation of Marzano’s taxonomy for General Chemistry, Example questions with learning objectives and Marzano categorization, Student Learning Objectives, Course grade structure, Data reduction methods, Rasch analysis including all attempts, Correlation of course score and first attempt score, and Minimal working example of the assessment codebase (PDF)

## References

[ref1] Havighurst R. J. Reform in the Chemistry Curriculum. J. Chem. Educ. 1929, 6, 1126–1129. 10.1021/ed006p1126.

[ref2] Lloyd B. W.; Spencer J. N. The Forum: New Directions for General Chemistry: Recommendations of the Task Force on the General Chemistry Curriculum. J. Chem. Educ. 1994, 71, 206 10.1021/ed071p206.

[ref3] Spencer J. N. New Directions in Teaching Chemistry: A Philosophical and Pedagogical Basis. J. Chem. Educ. 1999, 76, 566 10.1021/ed076p566.

[ref4] Cooper M. The Case for Reform of the Undergraduate General Chemistry Curriculum. J. Chem. Educ. 2010, 87, 231–232. 10.1021/ed800096m.

[ref5] Cooper M.; Klymkowsky M. Chemistry, Life, the Universe, and Everything: A New Approach to General Chemistry, and a Model for Curriculum Reform. J. Chem. Educ. 2013, 90, 1116–1122. 10.1021/ed300456y.

[ref6] Pazicni S.; Wink D. J.; Donovan A.; Conrad J. A.; Darr J. P.; Morgan Theall R. A.; Richter-Egger D. L.; Villalta-Cerdas A.; Walker D. R. The American Chemical Society General Chemistry Performance Expectations Project: From Task Force to Distributed Process for Implementing Multidimensional Learning. J. Chem. Educ. 2021, 98, 1112–1123. 10.1021/acs.jchemed.0c00986.

[ref7] Muñiz M. N.; Altinis-Kiraz C.; Emenike M. E. Extending Equity, Access, and Inclusion: An Evolving Multifaceted Approach to Transform a General Chemistry Course at a Large, Flagship, Research Institution. J. Chem. Educ. 2022, 99, 227–238. 10.1021/acs.jchemed.1c00387.

[ref8] Eberlein T.; Kampmeier J.; Minderhout V.; Moog R. S.; Platt T.; Varma-Nelson P.; White H. B. Pedagogies of Engagement in Science. Biochemistry and Molecular Biology Education 2008, 36, 262–273. 10.1002/bmb.20204. 19381266 PMC2665262

[ref9] Freeman S.; Eddy S. L.; McDonough M.; Smith M. K.; Okoroafor N.; Jordt H.; Wenderoth M. P. Active Learning Increases Student Performance in Science, Engineering, and Mathematics. Proc. Natl. Acad. Sci. U.S.A. 2014, 111, 8410–8415. 10.1073/pnas.1319030111. 24821756 PMC4060654

[ref10] Farrell J. J.; Moog R. S.; Spencer J. N. A Guided-Inquiry General Chemistry Course. J. Chem. Educ. 1999, 76, 570 10.1021/ed076p570.

[ref11] Moog R. S.; Spencer J. N. In Process Oriented Guided Inquiry Learning (POGIL); Moog R. S., Spencer J. N., Eds.; ACS Symposium Series; American Chemical Society: Washington, DC, 2008; Vol. 994; pp 1–13. 10.1021/bk-2008-0994.

[ref12] Lewis S. E.; Lewis J. E. Departing from Lectures: An Evaluation of a Peer-Led Guided Inquiry Alternative. J. Chem. Educ. 2005, 82, 135–139. 10.1021/ed082p135.

[ref13] Gosser D. K.; Roth V. The Workshop Chemistry Project: Peer-Led Team-Learning. J. Chem. Educ. 1998, 75, 185–187. 10.1021/ed075p185.

[ref14] Eilks I.; Byers B. Innovative Methods of Teaching and Learning Chemistry in Higher Education; Royal Society of Chemistry (Great Britain), 2009. 10.1039/9781839169212.

[ref15] Cooper M. M. Cooperative Learning: An Approach for Large Enrollment Courses. J. Chem. Educ. 1995, 72, 162–164. 10.1021/ed072p162.

[ref16] Roehling P. V. Creating and Implementing Effective Active Learning Experiences. Flipping the College Classroom 2018, 45–78. 10.1007/978-3-319-69392-7_3.

[ref17] Pavelich M. J.; Abraham M. R. An Inquiry Format Laboratory Program for General Chemistry. J. Chem. Educ. 1979, 56, 100–103. 10.1021/ed056p100.

[ref18] Deckert A. A.; Nestor L. P.; DiLullo D. An Example of a Guided-Inquiry, Collaborative Physical Chemistry Laboratory Course. J. Chem. Educ. 1998, 75, 860–863. 10.1021/ed075p860.

[ref19] Abrash S. A. Modern Developments in the Physical Chemistry Laboratory 2007, 973, 115–151. 10.1021/bk-2008-0973.ch008.

[ref20] Hunnicutt S. S.; Grushow A.; Whitnell R. Guided-Inquiry Experiments for Physical Chemistry: The POGIL-PCL Model. J. Chem. Educ. 2015, 92, 262–268. 10.1021/ed5003916.

[ref21] Marzano R. J. Formative Assessment & Standards-Based Grading; Solution Tree Press, 2009.

[ref22] Nilson L. B. Specifications Grading: Restoring Rigor, Motivating Students, and Saving Faculty Time; Stylus Publishing, LLC, 2015.

[ref23] Toledo S.; Dubas J. M. Encouraging Higher-Order Thinking in General Chemistry by Scaffolding Student Learning Using Marzano’s Taxonomy. J. Chem. Educ. 2016, 93, 64–69. 10.1021/acs.jchemed.5b00184.

[ref24] Toledo S.; Dubas J. M. A Learner-Centered Grading Method Focused on Reaching Proficiency with Course Learning Outcomes. J. Chem. Educ. 2017, 94, 1043–1050. 10.1021/acs.jchemed.6b00651.

[ref25] Clark D.; Talbert R. Grading for Growth: A Guide to Alternative Grading Practices that Promote Authentic Learning and Student Engagement in Higher Education, 1st ed.; Routlidge, 2023.

[ref26] Boesdorfer S. B.; Baldwin E.; Lieberum K. A. Emphasizing Learning: Using Standards-Based Grading in a Large Nonmajors’ General Chemistry Survey Course. J. Chem. Educ. 2018, 95, 1291–1300. 10.1021/acs.jchemed.8b00251.

[ref27] Martin L. J. ACS Symposium Series; 2019; Vol. 1330; Chapter 7, pp 105–119. 10.1021/bk-2019-1330.ch007.

[ref28] Howitz W. J.; McKnelly K. J.; Link R. D. Developing and Implementing a Specifications Grading System in an Organic Chemistry Laboratory Course. J. Chem. Educ. 2021, 98, 385–394. 10.1021/acs.jchemed.0c00450.

[ref29] McKnelly K. J.; Howitz W. J.; Thane T. A.; Link R. D. Specifications Grading at Scale: Improved Letter Grades and Grading-Related Interactions in a Course with over 1,000 Students. J. Chem. Educ. 2023, 100, 3179 10.1021/acs.jchemed.2c00740.

[ref30] Bunnell B.; LeBourgeois L.; Doble J.; Gute B.; Wainman J. W. Specifications-Based Grading Facilitates Student–Instructor Interactions in a Flipped-Format General Chemistry II Course. J. Chem. Educ. 2023, 100, 4318–4326. 10.1021/acs.jchemed.3c00473.

[ref31] Collins J. B.; Harsy A.; Hart J.; Anne Haymaker K.; Armstrong Hoofnagle A. M.; Kuyper Janssen M.; Stewart Kelly J.; Mohr A. T.; OShaughnessy J. Mastery-Based Testing in Undergraduate Mathematics Courses. PRIMUS 2019, 29, 441–460. 10.1080/10511970.2018.1488317.

[ref32] Kelly J. S. Mastering Your Sales Pitch: Selling Mastery Grading to Your Students and Yourself. PRIMUS 2020, 30, 979–994. 10.1080/10511970.2020.1733150.

[ref33] Marzano R. J.; Kendall J. S. The New Taxonomy of Educational Objectives, 2nd ed.; SAGE Publications, 2006.

[ref34] Stowe R. L.; Cooper M. M. Assessment in Chemistry Education. Isr. J. Chem. 2019, 59, 598–607. 10.1002/ijch.201900024.

[ref35] Moog R. S.; Webster G. H.; Farrell J. J. Chemistry: A Guided Inquiry, Part 1, 8th ed.; Kendall-Hunt, 2022.

[ref36] Vincent-Ruz P.; Meyer T.; Roe S. G.; Schunn C. D. Short-Term and Long-Term Effects of POGIL in a Large-Enrollment General Chemistry Course. J. Chem. Educ. 2020, 97, 1228–1238. 10.1021/acs.jchemed.9b01052.

[ref37] Flowers P.; Theopold K.; Langley R.; Robinson W. R. Chemistry, 2e; OpenStax: Houston, TX, 2019.

[ref38] ALEKS. https://www.aleks.com.

[ref39] TopHat. http://www.tophat.com, Accessed: 2023–09–19.

[ref40] Gradescope. http://www.gradescope.com, Accessed: 2023–09–19.

[ref41] Ralph V. R.; Scharlott L. J.; Schafer A. G.; Deshaye M. Y.; Becker N. M.; Stowe R. L. Advancing Equity in STEM: The Impact Assessment Design Has on Who Succeeds in Undergraduate Introductory Chemistry. JACS Au 2022, 2, 1869–1880. 10.1021/jacsau.2c00221. 36032534 PMC9400050

[ref42] Poore G. Reproducible Documents with PythonTeX 2013, 74–80. 10.25080/Majora-8b375195-00d.

[ref43] Poore G. M. PythonTeX: Reproducible Documents with LaTeX, Python, and More. Computational Science & Discovery 2015, 8, 014010 10.1088/1749-4699/8/1/014010.

[ref44] Hensel M. mhchem: v2021–12–31. 2021; https://ctan.org/pkg/mhchem.

[ref45] Tellechea C. chemfig: v1.6e. 2023; https://ctan.org/pkg/chemfig.

[ref46] Niederberger C. chemmacros: v6.2a. 2021; https://ctan.org/pkg/chemmacros.

[ref47] Niederberger C. modiagram: v0.3a. 2019; https://ctan.org/pkg/modiagram.

[ref48] Dahlgren B. ChemPy: A Package Useful for Chemistry Written in Python. Journal of Open Source Software 2018, 3, 565 10.21105/joss.00565.

[ref49] Ong S. P.; Richards W. D.; Jain A.; Hautier G.; Kocher M.; Cholia S.; Gunter D.; Chevrier V. L.; Persson K. A.; Ceder G. Python Materials Genomics (pymatgen): A robust, open-source python library for materials analysis. Comput. Mater. Sci. 2013, 68, 314–319. 10.1016/j.commatsci.2012.10.028.

[ref50] Kienzle P.; Pedersen B.; Forrester K.; Prescott S.; Juhas P.; Čermák P.; Dickinson M. pkienzle/periodictable: v1.6.0. 2021; https://github.com/pkienzle/periodictable.

[ref51] Schurmeier K. D.; Atwood C. H.; Shepler C. G.; Lautenschlager G. J. Using Item Response Theory to Assess Changes in Student Performance Based on Changes in Question Wording. J. Chem. Educ. 2010, 87, 1268–1272. 10.1021/ed100422c.

[ref52] Sorenson B.; Hanson K. Using Classical Test Theory and Rasch Modeling to Improve General Chemistry Exams on a per Instructor Basis. J. Chem. Educ. 2021, 98, 1529–1538. 10.1021/acs.jchemed.1c00164.

[ref53] Baker F. B.; Kim S.-H. The Basics of Item Response Theory Using R; Springer International Publishing: Cham, 2017. 10.1007/978-3-319-54205-8.

[ref54] Breakall J.; Randles C.; Tasker R. Development and Use of a Multiple-Choice Item Writing Flaws Evaluation Instrument in the Context of General Chemistry. Chemistry Education Research and Practice 2019, 20, 369–382. 10.1039/C8RP00262B.

[ref55] Press W. H. Numerical Recipes in C: The Art of Scientific Computing, 2nd ed.; Cambridge University Press: New York, 1997.

[ref56] Hunter R. A.; Pompano R. R.; Tuchler M. F. Alternative Assessment of Active Learning. ACS Symp. Ser. 2022, 1409, 269–295. 10.1021/bk-2022-1409.ch015.

[ref57] Ahlberg L. Organic Chemistry Core Competencies: Helping Students Engage Using Specifications. ACS Symp. Ser. 2021, 1378, 25–36. 10.1021/bk-2021-1378.ch003.

[ref58] Ralph V. R.; Lewis S. E. Impact of Representations in Assessments on Student Performance and Equity. J. Chem. Educ. 2020, 97, 603–615. 10.1021/acs.jchemed.9b01058.

[ref59] Santos D. L.; Gallo H.; Barbera J.; Mooring S. R. Student Perspectives on Chemistry Intelligence and their Implications for Measuring Chemistry-Specific Mindset. Chemistry Education Research and Practice 2021, 22, 905–922. 10.1039/D1RP00092F.

[ref60] Santos D. L.; Barbera J.; Mooring S. R. Development of the Chemistry Mindset Instrument (CheMI) for use with introductory undergraduate chemistry students. Chemistry Education Research and Practice 2022, 23, 742–757. 10.1039/D2RP00102K.

[ref61] Fink A.; Cahill M. J.; McDaniel M. A.; Hoffman A.; Frey R. F. Improving General Chemistry Performance through a Growth Mindset Intervention: Selective Effects on Underrepresented Minorities. Chemistry Education Research and Practice 2018, 19, 783–806. 10.1039/C7RP00244K.

[ref62] Limeri L. B.; Carter N. T.; Lyra F.; Martin J.; Mastronardo H.; Patel J.; Dolan E. L. Undergraduate Lay Theories of Abilities: Mindset, Universality, and Brilliance Beliefs Uniquely Predict Undergraduate Educational Outcomes. CBE Life Sciences Education 2023, 22. 10.1187/cbe.22-12-0250. PMC10756031 37751502

[ref63] Currie H. N. Mindful Well-Being and Learning. J. Chem. Educ. 2020, 97, 2393–2396. 10.1021/acs.jchemed.0c00777.

[ref64] Lewis D. Impacts of Standards-Based Grading on Students’ Mindset and Test Anxiety. Journal of the Scholarship of Teaching and Learning 2022, 22. 10.14434/josotl.v22i2.31308.

[ref65] Feldman J. Grading for Equity: What It Is, Why It Matters, and How It Can. Transform Schools and Classrooms; Corwin Press: Thousand Oaks, CA, 2018.

[ref66] Shah L.; Fatima A.; Syed A.; Glasser E. Investigating the Impact of Assessment Practices on the Performance of Students Perceived to Be at Risk of Failure in Second-Semester General Chemistry. J. Chem. Educ. 2022, 99, 14–24. 10.1021/acs.jchemed.0c01463.

[ref67] White K. N.; Vincent-Layton K.; Villarreal B. Equitable and Inclusive Practices Designed to Reduce Equity Gaps in Undergraduate Chemistry Courses. J. Chem. Educ. 2021, 98, 330–339. 10.1021/acs.jchemed.0c01094.

[ref68] Harris R. B.; Mack M. R.; Bryant J.; Theobald E. J.; Freeman S. Reducing achievement gaps in undergraduate general chemistry could lift underrepresented students into a “hyperpersistent zone. Science Advances 2020, 6. 10.1126/sciadv.aaz5687. PMC7286681 32577510

