# Supplementary material for: Low-Stakes, Growth-Oriented Testing in Large-Enrollment General Chemistry 1: Formulation, Implementation, and Statistical Analysis
Source: J Chem Educ. 2024 Jul 25;101(8):3097–106. doi: 10.1021/acs.jchemed.3c00993 (PMC11328131; doi:10.1021/acs.jchemed.3c00993)
Supplement: Supplementary file 1 — ed3c00993_si_001.pdf [file ed3c00993_si_001.pdf]

Supporting Information: Low-Stakes, Growth-Oriented Testing in Large-Enrollment General Chemistry 1: Formulation, Implementation, and Statistical Analysis

Tricia D. Shepherd* and Sean Garrett-Roe*

Department of Chemistry, University of Pittsburgh, Pittsburgh, PA, USA

E-mail: tds53@pitt.edu; sgr@pitt.edu

S1 Marzano's Taxonomy Interpreted in the General Chemistry Context

S1.1 Interpretation of Marzano's Levels

The following discussion expands on how we operationalized the four levels of Marzano's taxonomy using examples from Knowledge Focus 1.1 (Atomic structure). Figure 1, center, lists the aligned learning objectives.

Retrieval describes innate mental processes generally done without conscious awareness. Information is simply remembered or recognized, and procedures are executed automatically. "There is no expectation that a student will know the knowledge in depth, be able to identify the basic structures of the knowledge (or its critical verses noncritical elements), or use it to accomplish complex goals." (Marzano and Kendall[1], p. 65). As our working definition of

retrieval, a student will be able to:

> **Retrieval Level:** Recognize and recall basic information and execute simple, algorithmic procedures.

Example learning objectives are given in Figure 1, center, R1–R3. A simple algorithmic procedure, like converting grams to moles (R2), is categorized as a *retrieval* level objective. (More precisely, it is an execution task, which is a sub-category of the retrieval level. For operational simplicity, we do not distinguish the sub-levels.) These calculations have very specific, unvarying steps. "Algorithms must be learned to a level of automaticity to be useful" (Marzano and Kendall[1], p. 13), and they are common kinds of tasks in General Chemistry. These skills are not inherently *retrieval*, but, through learning and practice, students are able to achieve a *retrieval* proficiency at some point during the General Chemistry course. Typically, the calculations at the *retrieval* level have few steps (one or two), as illustrated by specific questions.

The next level, *comprehension*, requires more conscious thought from students. Students are expected to understand the basic structure of the knowledge and control its critical or defining attributes. A student will be able to

> **Comprehension:** Accurately represent information in symbols, graphs, and equations and use that information to indicate and explain relationships or reason qualitatively.

Typical learning objectives at the *comprehension* level are given in Figure 1, center, C1 and C2. Translating information from a symbolic form (the nuclide symbol) to its essential facts (the number of protons, neutrons, and electrons), and transforming the symbolic form to or from a particulate representation (C2) are characteristic of a major intellectual challenge of General Chemistry for students. Chemists switch between many kinds of representations as suits our needs – atomic/molecular, macroscopic, and symbolic forms. Students must master

the interrelations of these representations, which makes *comprehension* more cognitively demanding than *retrieval*.

Analysis and *knowledge utilization* are higher-level objectives. Our working definitions are that a student can

> **Analysis:** Apply concepts to solve problems with multiple correct approaches, identify errors, predict new trends, compare and contrast, and explain their reasoning.

and

> **Knowledge Utilization:** Use knowledge creatively in a new context and justify their approach.

Both levels can involve problem solving, which should have a novel aspect – students should be required to use the facts and algorithms they know (*retrieval*) and their understanding of how they interrelate (*comprehension*) to devise a solution to the stated problem. *Analysis* questions, however, typically have a single correct answer, though several potential routes may be available to arrive to it. *Knowledge Utilization* questions, on the other hand, are distinct from *analysis*, because many answers are correct, not just one.

We use the term "problem solving" to mean mental activity that requires new conceptual connections among the ideas in the learning objective. We distinguish this from answering word problems that require a sequence of rote steps, perhaps in a confusing or distracting presentation. In these types of word problems, the analysis that occurs is focused on interpreting the question rather than analyzing the chemical data or concepts.

The cognitive complexity in *knowledge utilization* questions derives from the requirement to propose or decide and, then, to justify the choices. *Knowledge Utilization* questions often provide a context and ask students to solve a challenge in that context. We primarily aligned learning objectives to Marzano's "Decision-Making" and "Problem-Solving Objectives." The "Experimenting" and "Investigating" objectives are out of scope for brief, in-class assessments.

Both *analysis* and *knowledge utilization* questions are framed by the context of the *retrieval* and *comprehension* questions in their Knowledge Focus. The two higher level questions may provide opportunities for students to connect ideas from otherwise distinct learning objectives.

A student's real mental experience completing a task depends on all of their prior experiences. To an expert, complex procedures can be executed with little mental effort (*retrieval*), while a simple task may require the creation of new understanding from a novice (*analysis*). Does this relativity mean the process of categorizing learning objectives and assessment items is hopeless?

We argue no. We categorize learning objectives and items in the four levels based on the learning experiences that we target for our students. *Retrieval* and *comprehension* tasks involve processes and ideas to which students have been exposed multiple times. We construct the in-class activities, readings, homework, and recitation problems to reinforce these fundamentals. *Analysis* and *knowledge utilization*, however, require novelty. Asking students to answer the same *analysis* question more than once would give them an opportunity to memorize the algorithm. The lack of novelty could fundamentally change the mental processes involved and transform the item into a *retrieval* question. As a result, we control which assessment items students are exposed to and ensure they get novel questions at the *analysis* and *knowledge utilization* levels in particular. Instructors *must* "teach to the test" at the *retrieval* and *comprehension* levels, because the student must have been exposed to the material; the instructor *cannot* teach to the test at the *analysis* or *knowledge utilization* levels, because, if they do, the questions are neither *analysis* nor *knowledge utilization*.

Our system aligns well with other Depth-of-Knowledge categorizations. For example, Webb[2] proposes very similar strata. The largest difference is that our *knowledge utilization* level emphasizes creativity in generating responses that have more than one correct response whereas Webb emphasizes the extended length of time for a complex task (which we must exclude).

A recent effort to define conceptual understanding proposed its five essential aspects based on the input of ~ 1500 instructors.[3] All of these aspects map to levels of Marzano's taxonomy, roughly. "A student who demonstrates conceptual understanding can: 1. **(Transfer)** Apply core chemistry ideas to situations that are novel to the student, 2. **(Depth)** Reason about core chemistry ideas using skills that go beyond rote memorization and or algorithmic problem solving, 3. **(Predict/Explain)** Expand situational knowledge to predict and/or explain chemical behavior, 4. **(Problem Solving)** Demonstrate the critical thinking and reasoning involved in solving problems including laboratory measurement, 5. **(Translate)** Translate across scales and representations."[3] In our framework, statement 2 (Depth) could fit any of the categories other than *retrieval*, depending on the kind of reasoning required. Statement 5 (Translate) is clearly *comprehension*, involving symbolizing and integrating. Statements 3 (Predict/Explain) and 4 (Problem Solving), align to *analysis* tasks, though if the required explanation is of well-established phenomena (to the student) then the task is more *comprehension*. Statement 1 (Transfer) would likely map to a *knowledge utilization* task, given the emphasis on using knowledge in a novel context, provided that the range of acceptable solutions was open-ended; were only a single answer considered correct, this would likely be an *analysis* task. All of these aspects of conceptual understanding outlined by Holme et al.[3] are incorporated, clarified, and organized in our hierarchy.

S1.2 Interpretation of Marzano's Verbs

Marzano summarizes the cognitive levels of the taxonomy with a list of verbs (Table S1).

In applying this taxonomy in General Chemistry, however, several modifications proved necessary. First and foremost is the observation that individual verbs are inadequate to determine the level of a question. Far more important is the context and the mental process needed to take the inputs and reach the requested outputs. The same verb can correspond to different levels. For example, we use "determine" in questions at *retrieval*, *comprehension*, and *analysis* levels. At the *retrieval* level, we identify two classes of determination, one

Table S1: The original list of verbs at the levels of Marzano's taxonomy.

1. Retrieval:
    - Recognizing: select or identify from a list, determine true/false
    - Recalling: name, list, state, identify fact, describe what
    - Executing: verify, complete, demonstrate
2. Comprehension:
    - Integrating: summarize, describe relationships, describe how, why, effects
    - Symbolizing: Use models to represent, depict, draw, illustrate, diagram, chart
3. Analysis: Apply concepts to solve problems with multiple correct approaches, identify errors, predict new trends, compare and contrast, and explain your reasoning
    - Matching: compare/contrast, categorize, sort, distinguish, create analogy
    - Classifying: organize, sort
    - Analyze errors: evaluate, identify errors, revise, critique
    - Generalizing: form conclusion or inference, create rule/principle
    - Specify: predict, justify, judge, deduce
4. Knowledge Utilization: Use knowledge creatively in a new context and justify your approach
    - Decision Making: decide between alternatives and provide a justification for why the chosen approach is optimal
    - Problem Solving: develop creative solutions to problems
    - Experimenting: explore with new approaches or existing approaches with new systems
    - Investigating: propose new directions to explore with a rationale for why

qualitative and one quantitative. The qualitative determination is illustrated by, for example, determine if a reaction is endothermic or exothermic based on the sign of ΔH_{rxn}; at the *comprehension* level, it can be to determine the ΔH_{rxn} based on an enthalpy diagram; at the analysis level, it can be to determine outcome in new (novel) scenario and explain the answer (sensemaking).

"Calculate," "identify," and "determine" are all verbs that appear at multiple levels, especially *retrieval* and *analysis*. Defining differences are that, at the *retrieval* level, the calculation occurs in one or two steps which have been presented before to students, while, at the *analysis* level, the calculation may have multiple steps, which could be completed in different orders, and should have novel aspect that students have not seen before, which could be either the inputs or the outputs.

At the Comprehension level, we identified three aspects of "Integrating" information, which Marzano explains as "Students identify the basic structure of knowledge and the critical as opposed to noncritical characteristics." In itself, this statement did not help us to write or classify questions. We do identify three broad classes of question which do meet some of the criteria for "integrating."

The verb most uniquely associated with a level, in our implementation, is "propose," which we associate with the *knowledge utilization* level almost exclusively. The essential quality is that many proposals could satisfy the requirements. In almost all cases, *knowledge utilization* questions are open-ended. In contrast, *analysis* questions provide specific, concrete constraints that limit the answers to a specific correct outcome.

In our learning objectives, we use the following categories and typical verbs (Table 2).

Table S2: The set of verbs used to assign questions to levels of Marzano's taxonomy with some examples.

1. Retrieval: Recognize and recall basic information and execute simple, algorithmic procedures
   - **Recognizing:** Identify familiar information (identify, select, choose)
     - Select atoms or molecules based on direct application of known properties or trends (e.g., select most / least electronegative atom, highest/lowest ionization energy, etc.)
   - **Recalling:** Use known trends or information to reach unambiguous conclusions (determine, identify, rank)
     - Determine qualitative categorization from definitions and sufficient information (e.g., endo/exothermic, sign of ΔH, soluble/insoluble, etc.),
     - Identify unambiguous atomic, molecular, or macroscopic properties (names, core charge, quantum numbers, etc.),
     - Rank by known properties or trends (e.g., most/least electronegative, highest/lowest ionization energy)
   - **Executing:** Perform calculations or known procedures in a few steps (calculate, convert, balance, determine, use)
     - Convert quantities (number / moles / grams / concentration / volume),
     - Calculate using known formulas (e.g., ideal gas equation, r.m.s. velocity, effusion rates, etc.)

- Use rules (e.g., the solubility rules),
- Balance a chemical reaction,
- Determine quantitative properties using known principles and relationships (e.g., what is the mass / volume / molarity, how many atoms / grams / moles, what is the empirical formula/percent mass, change in enthalpy, what is the specific heat/heat capacity, magnitude of attractive / repulsive forces between charges, frequency / wavelength / energy, ionization energy / kinetic energy / binding energy / photon energy, formal charge, bond order, density, oxidation numbers)

2. Comprehension: Accurately represent information in symbols, graphs, and equations and use that information to indicate and explain relationships or reason qualitatively

   - **Integrating:** Extract and use meaning and determine relationships from symbolic or graphical information (use, predict, set up, interpret)
     - Use representations to establish relationships and explain reasoning (e.g., use electronegativity data and molecular structure to determine most/least polar bond; use MO diagram to support reasoning about bond order, magnetic moment; use Lewis structures to rank/compare dipole moment; draw dipole and use it to explain polarity; use molecular diagrams to explain relative intermolecular forces),
     - Use qualitative reasoning based on quantitative principles to draw conclusions and support reasoning (e.g., predicting direction / amount heat flow or or sign / magnitude of ΔT from observations or comparing systems, applying Hund's rule or the Pauli exclusion principle, calculate enthalpy of reaction from qualitative data and interpret endo/exothermic, etc.)
     - Manipulate or transform abstract representations (e.g., use Hess's Law with multiple chemical equations, set up a calorimetric equation, write balanced molecular equation / net ionic equation from description of a reaction)
   - **Symbolizing:** Encode, decode, and translate information in symbolic forms (draw, write, identify, calculate, sketch, interpret)
     - Draw a graphical representation based on atomic or molecular information (a particulate representation of atom, reaction, or solution, an energy level or enthalpy diagram corresponding to data, isosurfaces / radial distribution plots of related atoms, Molecular Orbitals from given Atomic Orbitals, the best Lewis structure, phase diagrams)
     - Identify the information about an atom or molecule based on a symbolic or graphical representation (e.g., a nuclide symbol from numbers of protons, neutrons, and electrons, an element from photoelectron spectrum, key properties from a chemical formula or reaction, atoms/ions with a certain valence shell configuration, hybridization/bond angle in molecule, etc.)

- Predict atomic and molecular properties from drawings (bond order from Lewis structure)
- Construct an appropriate diagram to support a conclusion or reasoning.

3. Analysis: Apply concepts to solve problems with multiple correct approaches, identify errors, predict new trends, and explain reasoning or justify approach
   - **Problem solving 1 (Specifying):** (predict, justify, determine, use, calculate)
     - Calculate a specific outcome when several steps are required and at least one step or aspect (inputs/givens or outputs) is novel (many typical, challenging word problems are in this category provided they include a "twist", a new connection between concepts or a new aspect of the information)
     - Determine the outcome in new (novel) scenario, and make sense of the answer (e.g., consequences of new rules for quantum numbers)
   - **Generalizing:** form conclusion or inference from data, create rule/principle from specific evidence (propose)
   - **Analyzing errors:** (evaluate, identify potential errors)
4. Knowledge Utilization: Use knowledge creatively in a new context and justify your approach
   - **Decision making:** Decide between alternatives and provide a justification for why the chosen approach is optimal
   - **Problem solving 2:** Develop creative solutions to problems with many correct solutions

S1.3 Characterization of Assessment Items

The $\sim$ 300 questions in the question bank utilize a variety of question types, the vast majority of which are open-ended (Table S3). We categorize our question types as follows

Multiple choice A student selects one correct answer from a small set of options, typically 4 to 6.

Multiple selection A student selects all terms that apply; more than one selection may be correct.

Fill-in-the-blank A student provides requested information in several categories (e.g., Sec 2.1 Example 1).

Calculation A student calculates a value (e.g., Sec 2.1 Example 2).

Table S3: Question Stems by Marzano Level Coded by Question Type and Math versus Mechanistic Reasoning

	R	C	A	U	total
Multiple choice	6	1			7
Multiple selection	3	7			10
Fill-in-the-blank	28	13	3		44
Calculation	54	3	56	18	131
Explanation	1	19	25	53	98
Symbolic	6	12		1	19
Drawing	1	30	13	22	66
Integrated	1	11	15	32	59
Math	54	8	50	18	130
Mechanistic	1	40	27	55	123
Both	0	4	5	13	22
Neither	43	28	3	0	74
Total	98	72	75	60	305

Explanation A student explains a phenomenon (e.g., Sec 2.4 Example 6).

Symbolic A student provides an appropriate symbolic response, such as a nuclide symbol, a chemical formula, or a chemical equation (e.g., Sec 2.2 Example 3).

Drawing A student draws a diagram, illustration, or figure (e.g., Sec 2.2 Example 4).

Integrated A student must integrate multiple answer types, which are usually an explanation along with a calculation, drawing, or symbol (e.g., Sec 2.3 Example 5).

All question types except multiple choice and multiple selection are free response questions.

Only a small fraction of questions stems in the bank are multiple-choice (2 %); a similar fraction (3 %) are multiple selection questions. Nearly all questions request student generated responses in the forms of calculations, drawings, and symbols (especially atomic symbols, chemical formulas, and chemical equations). Many questions prompt for an explanation (32 %) and a sizeable fraction (19 %) require an integrated response of an explanation with a drawing, symbol, or calculation.

Ralph et al.[4] have discussed the impact that different question styles can have on the performance of students from underrepresented minority groups. They recommend that General Chemistry assessments increase the fraction of questions that elicit mechanistic reasoning. They code questions as “math” or “mechanistic reasoning”.

For comparison, we have coded the 305 question stems in our question bank with the “Math” and “Mechanistic Reasoning” codes suggested by Ralph et al. sorted by their level in Marzano’s taxonomy (Table S3). “Math” reasoning (in the sense of Ralph et al.) is reasoning based on multiplicative, proportional, or functional reasoning (i.e., math beyond counting skills like addition and subtraction). “Mechanistic reasoning” consists of an explanation of a phenomenon using underlying causes or making predictions based on those causes. Only one of 98 questions at the *retrieval* level (R) elicits mechanistic reasoning; this was not our design goal but fits entirely within our framework. *Comprehension*, on the other hand, requires mechanistic reasoning to a large degree ($40/72 = 56\,\%$) because these items ask students to explain their understanding of the phenomenon in terms of underlying models. *Analysis* questions typically request students to justify their answers, and in many cases a mathematical analysis (calculation) may suffice, hence a large fraction of questions are coded “Mathematical” ($50/75 = 67\,\%$); nevertheless, in a substantial fraction of *analysis* questions ($27/75 = 36\,\%$) students must articulate correct mechanistic reasoning. *Knowledge Utilization* questions (U) require mechanistic reasoning overwhelmingly ($55/60 = 92\,\%$). Again, mechanistic reasoning was not our design goal for this level, but nearly all of our *knowledge utilization* questions require clearly articulated mechanistic reasoning to be proficient.

Our assessment system presents students with “mechanistic reasoning” tasks above the rate of the “Curriculum Reform” of Ralph et al. and “math” tasks at a similar rate. The reformed curriculum of Ralph et al. included $30\,\%$ mechanistic reasoning tasks and $30/92 = 33\,\%$ math tasks in General Chemistry 1. Because we randomize assessment questions, we must characterize our assessments statistically. Based on the statistics of questions in each Marzano level, the expected number of mechanistic questions (out of 4 total) on any

particular assessment is 1.9 ± 0.8. Students have a 98 % chance of the four question assessment containing 1 or more mechanistic reasoning tasks; 68 % of 2 or more; 19 % of 3 or more. As a result, roughly half of the tasks we present a student require mechanistic reasoning. The expected number of math tasks is 1.6 ± 0.9 (again out of 4 total), roughly 40 %.

In closing, we emphasize that *knowledge utilization* and "mechanistic reasoning" are *not* synonyms. The exemplar mechanistic questions in Ralph et al.[4], Figures 2 and 3, are *comprehension* questions in multiple-choice format. We cannot envision a close-ended, multiple choice question which could be a *Knowledge Utilization* question. We require that *Knowledge Utilization* questions elicit *creative* solutions to *novel* scenarios in which more than one answer is correct (cf., Supporting Information Sections 1 and 2.4).

S2 Example Learning Objectives and Assessment Items for Knowledge Focus 1.1

Examples of assessment items and their aligned student learning objectives are given for Knowledge Focus 1.1. A statement from Marzano's taxonomy that justifies the alignment in the taxonomy is also provided for each question. The programmatically determined quantities are highlighted in cyan boxes. The solution and grading notes are also presented.

S2.1 *Retrieval*

Retrieval: Recognize and recall basic information and execute simple, algorithmic procedures

- **R1** Identify the number of protons, neutrons, electrons, mass number, atomic number, and overall charge of an atom (any isotope or ion)
- **R2** Convert between number of atoms, grams, and moles of an element
- **R3** Calculate the average atomic mass of an element given its isotopes and their natural abundance

Marzano statement: "When asked about specific details, the student produces related information."

Example 1. (R1) (difficulty = −1.61, point-biserial correlation = 0.42, $n = 34$)

Identify the following for an isotope of germanium with a mass number of 74 and 32 electrons.

a) the number of protons: 32
b) the number of neutrons: 42
c) the charge: 0

Solution: Grading Note: Minor error only if clear math error (e.g., $52 - 24 = 22$). The sign of the charge must be correct.

Marzano statement: "The student performs a procedure without significant error."

Example 2. (R2) (difficulty $= -1.37$, point-biserial correlation $= 0.43$, $n = 64$)

How many helium atoms are in 9.35 g of helium?

Answer: 1.41×10^{24} atoms

Solution:

$$\frac{9.35\ \cancel{\text{g}}}{1} \; \frac{1\ \cancel{\text{mol}}}{4.003\ \cancel{\text{g}}} \; \frac{6.02 \times 10^{23}\ \text{atoms}}{1\ \cancel{\text{mol}}} = 1.41 \times 10^{24}\ \text{atoms}$$

Grading Note: Answer is proficient if the work shown is correct.

S2.2 *Comprehension*

Comprehension: Accurately represent information in symbols, graphs, and equations and use that information to indicate and explain relationships or reason qualitatively

C1 Write or interpret nuclide symbols to represent an element, ion, or isotope.

C2 Sketch or interpret a particulate representation of an element, ion, or isotope

Marzano statement: "The student accurately represents the major aspects of details in nonlinguistic or abstract form"

Example 3. (C1) (difficulty = −1.17, point-biserial correlation = 0.42, $n = 125$)

Write the nuclide symbol of an element that has the same number of neutrons as ^{20}Ne, but a different mass number.

Solution: Any element X for which its atomic number + 10 ≠ 20. Final answer should be written in form: $^{(\text{atomic number}+10)}\text{X}$.

Grading Note: A proficient answer must identify a different element symbol than Ne. If no work shown, mass number must be correct. If answer identities correct number of neutrons, but mass number is off by one or two, assume minor math error.

Example 4. (C2) (difficulty = 0.31, point-biserial correlation = 0.40, $n = 86$)

Make a drawing (and brief explanation if desired) that clearly illustrates what is represented by the following symbol: $^{1}\text{H}^{2-}$.

Solution: Drawing with 1 protons and 0 neutrons in the nucleus and 3 electrons outside the nucleus. (Like ChemActivity 1 Model 1)

Grading Note: A proficient answer must represent protons, neutrons, and electrons as particles (Not as letters or element symbol). Proton and neutron particles should be in the center (nucleus) and electrons should be outside spaced apart (ignore any shell designation). Must have correct number of protons and electrons. Minor error if number of neutrons is incorrect.

S2.3 *Analysis*

Analysis: Apply concepts to solve problems with multiple correct approaches, identify errors, predict new trends, compare and contrast, and explain your reasoning

A1 Solve problems with multiple correct approaches involving average mass, numbers of atoms, or moles of an element.

Marzano statement: "The student constructs and defends new generalizations and principles based on known details."

Example 5. (A1) (difficulty = 0.72, point-biserial correlation = 0.24, $n = 76$)

What would be the value of Avogadro's number if a mole were defined as the number of ^{14}N atoms in 0.267 mg of ^{14}N atoms? Explain whether or not your answer seems reasonable.

Answer: 1.15×10^{19}

Solution: The answer will be greater or less than 6.02×10^{23} based on how the new definition's mass (in this case 0.267 mg) and molar mass (^{14}N: 14 g mol^{-1}) compares to the old definition's mass (12 g) and the atomic mass of ^{12}C (exactly 12.0 g mol^{-1}). The new Avogadro number is smaller as the new mass is smaller than 12.0 g and the molar mass is heavier than 12.0 g mol^{-1}. The new number is

$$0.267 \frac{1\,\text{g}}{1000\,\text{mg}} \frac{1\,\text{mol}}{14\,\text{g mol}^{-1}} \frac{6.02 \times 10^{23}}{1\,\text{mol}} = 1.15 \times 10^{19}$$

S2.4 *Knowledge Utilization*

Knowledge Utilization: Use knowledge creatively in a new context and justify your approach

U1 Propose a solution to a problem using mass, moles, and number of atoms and provide appropriate justifications for all quantities identified.

Marzano statement: "The student uses his or her knowledge of details to solve a specific problem."

Example 6. (U1) (difficulty = 1.0, point-biserial correlation = 0.37, $n = 51$)

Metal clusters are groups of bound atoms that tend to form with certain "magic numbers" (*e.g.*, 2, 8, 18, 20, 34, 40, 58, 92...). If you know the number of paladium clusters in a sample, propose how you would determine the number of atoms in the cluster. What additional information would be needed to make this determination? Explain.

Solution:

Student Version: Answer describes how to obtain the number of atoms in a cluster using the known quantity (number of clusters in a sample). All quantities are associated with a specific item (e.g., mass of a single atom, mass of the cluster, number of atoms in the sample...) and a reasonable way of either measuring or calculating the quantity used to solve the problem. (e.g., there is no scale that can measure the mass of a cluster of atoms.)

Detailed Solution: Measure the mass of the sample. Divide the sample mass by the number of clusters to determine the mass of a cluster. Once you know the cluster mass, convert to moles using the atomic mass of the metal and, then, number of atoms with Avogadro's number. Or covert the total sample mass to atoms and divide by the number of clusters.

S3 Student Learning Objectives for General Chemistry 1

Unit 1: Atoms, compounds, and chemical equations

Knowledge Focus 1.1: Atoms and moles

R1: Identify the number of protons, neutrons, electrons, mass number, atomic number, and overall charge of an atom (any isotope or ion)

R2: Convert between number of atoms, grams, and moles of an element

R3: Calculate the average atomic mass of an element given its isotopes and their natural abundance

C1: Write or interpret nuclide symbols to represent an element, ion, or isotope

C2: Sketch or interpret a particulate representation of an element, ion, or isotope

A1: Solve problems with multiple correct approaches involving average mass, numbers of atoms, or moles of an element

U1: Propose a solution to a novel problem involving mass, moles, and/or number of atoms and provide appropriate justifications for all quantities identified

Knowledge Focus 1.2: Compounds

R1: Identify the name or formula of binary molecular, ionic and polyatomic compounds

R2: Convert between number (molecules or atoms), moles, grams of a compound or component

R3: Convert between a molecular formula of a compound and its percent composition by mass

C1: Sketch or interpret a particulate representation of atomic, ionic, or molecular species including phase (solid, liquid, gas)

A1: Solve problems with multiple correct approaches involving the names, quantities and composition of a compound

U1: Propose a solution to a novel problem involving the names, quantities and composition of a compound

Knowledge Focus 1.3: Chemical equations and solutions

R1: Identify stoichiometric coefficients and calculate quantities of species associated with a balanced chemical equation

R2: Calculate the percent yield of a reaction

R3: Calculate the molarity of a solution and use concentration to determine quantities involving moles, mass or volume

C1: Construct symbolic or particulate representations of a chemical reaction and determine quantities in the presence of a limiting reactant

A1: Solve problems with multiple correct approaches involving chemical reactions and solutions

U1: Describe an appropriate procedure to make a solution based on available materials and intended use

U2: Propose chemical reaction conditions in the presence of a limiting reactant consistent with the observations described

Unit 2: Chemical reactions and energy changes

Knowledge Focus 2.1: Reaction types

R1: Use the solubility rules to determine all major species present in a solution

R2: Assign oxidation numbers to atoms, ionic and molecular compounds, and ions

C1: Sketch particulate or interpret symbolic representations of a solution

C2: Determine whether an element is oxidized or reduced, the oxidizing and reducing agents, and the number of electrons transferred in a redox reaction

C3: Write balanced net ionic equations and identify spectator ions

A1: Identify all species present and their amount when combining multiple solutions

A2: Predict possible chemical reactions and solve quantitative problems with multiple correct approaches

U1: Propose a solution to a problem involving aqueous or redox reactions providing appropriate justification of any choices made

Knowledge Focus 2.2: Enthalpy

R1: Define enthalpy of atom combination, exothermic, and endothermic based on energy gain or loss and bond making or breaking processes

R2: Calculate reaction enthalpies based on the amounts and enthalpies of atom combination of reactants and products

C1: Use enthalpy of atom combination data to sketch or interpret an energy diagram and calculate standard reaction enthalpies

C2: Determine if a reaction is endothermic or exothermic and compare relative bond strengths based on an energy diagram or enthalpy of atom combination data

A1: Predict possible trends and solve quantitative problems with multiple correct approaches using enthalpies of atom combination

U1: Use the concepts of enthalpy to evaluate, compare, and or predict the energetics of chemical reactions

Knowledge Focus 2.3: Thermochemistry

R1: Define relationships and calculate quantities involving heat, heat capacity, mass, temperature

R2: Identify and calculate changes of temperature and enthalpy for endothermic or exothermic processes based on amount of heat transferred

R3: Define enthalpy of formation and calculate reaction enthalpies based on the enthalpies of formation of reactants and products

C1: Use Hess's Law to determine the enthalpy change of a reaction

C2: Make qualitative predictions about heat flow based on mass, heat capacity, temperature, and reaction enthalpies

A1: Solve problems with multiple correct approaches involving the transfer of heat and enthalpy changes of reaction

U1: Propose a solution to a problem involving heat, temperature, and enthalpy providing appropriate justification of any choices made

Unit 3: Atomic models and electronic structure

Knowledge Focus 3.1: Coulomb's law, shell model, and periodic trends

R1: Define Coulomb's potential energy equation and relate its value to higher/lower force of attraction/ repulsion between two particles

R2: Identify the core charge, valence shell, number of core and valence electrons for an atom

C1: Construct diagrams of an atom or ion using the shell model and core charge concept. Use these diagrams to compare core charges, ionization energies, and radii

C2: Explain general trends in ionization energy, radius, core charge, valence shell, number of core and valence electrons for atoms and ions

A1: Make predictions and solve problems with multiple correct approaches involving atomic and ionic properties using the shell model

U1: Propose a method to assess ionization energies or other relationships involving charged particles and justify the approach using Coulomb's Law

Knowledge Focus 3.2: Photoelectron spectroscopy and electron configurations

R1: Define basic properties of electromagnetic radiation and convert between frequency, wavelength (nm), and energy (J or kJ/mol)

R2: Recognize and convert between various types of energy for an electron based on the: energy level, ionization energy, photon energy, and kinetic energy

C1: Sketch or interpret the key features of the photoelectron spectra and how they relate to the shell model, energy level diagram, or electron configuration of an atom or ion

C2: Write or interpret a ground or excited state electron configuration for any element

A1: Based on energy conservation principles, solve problems involving the energy of a photon, ionization energy and kinetic energy of an electron

U1: Solve a novel photoemission problem, propose any needed quantities or conditions, and justify your choices

Knowledge Focus 3.3: Electron spin, orbitals, and quantum numbers

R1: Identify a valid set or subset of quantum numbers (n, l, m_l, m_s) and what these numbers represent about an electron including the number of angular and radial nodes

R2: Identify and calculate the wavelengths of absorption and emission electronic transitions based on the Bohr model

C1: Sketch or interpret ground or excited state orbital diagrams comparing magnetic moments and following Hund's rule and the Pauli exclusion principle

C2: Sketch or interpret radial distribution or isosurface plots of atomic orbitals including radial and angular nodes

A1: Solve problems involving energies or configurations of ground and excited electronic states

U1: Propose a solution to a problem involving energies or configurations of ground and excited electronic states providing appropriate justification of any choices made

Unit 4: The nature of chemical bonding

Knowledge Focus 4.1: Lewis structures, resonance, and formal charge

R1: Determine the total number of valence electrons, covalent bonds, and lone electron pairs for a given molecule

R2: Determine the formal charge of each atom and bond orders for a given Lewis structure

C1: Sketch all valid Lewis structures and interpret resonance effects involving formal charge, bond order, and bond length

A1: Make predictions and analyze properties of the 'best' Lewis structure(s) based on formal charge and expanded octets

U1: Propose a particular arrangement of elements that would most likely form a molecule

Knowledge Focus 4.2: Molecular orbital theory, hybridization, and molecular shape

R1: Identify relationships between the number of bonding domains, number of nonbonding domains, electron geometry, molecular geometry, orbital hybridization, and bond angles

C1: Sketch or describe the region of space atoms/electrons occupy in molecules based on VSEPR, molecular orbital, or valence bond theory

A1: Compare the properties of molecules (e.g., spin, bond order, π- and σ-bonds, bond angles, bond length, bond strength) using an appropriate bonding theory

U1: Apply an appropriate bonding theory in a new context

Knowledge Focus 4.3: Polarity, electronegativity, and partial charge

R1: Identify the most electronegative element use this information to determine which element has a positive or negative partial charge

R2: Calculate partial charge from electronegativity

C1: Determine appropriate partial charges (sign or magnitude) of an atom and describe whether the bond is nonpolar, polar, or ionic

A1: Compare the magnitude and direction of individual bond dipoles and molecular dipole moments

U1: Compare a series of related molecules in terms of bond and molecular polarity and determine which molecule would best exhibit coulombic interactions necessary for a desired property or application

Unit 5: Phases of matter

Knowledge Focus 5.1: Kinetic molecular theory, intermolecular forces, and phase diagrams

R1: Use the ideal gas law to calculate P, V, T, or n given the other quantities

R2: Calculate r.m.s. velocity of a molecule given its temperature and vice versa

R3: Compare gas densities at a particular temperature and pressure

C1: Sketch the Maxwell-Boltzmann speed distribution and describe changes with mass or temperature

C2: Identify and illustrate the type of intermolecular forces associated with a given molecule

A1: Solve quantitative problems involving the ideal gases with multiple correct approaches

A2: Make predictions and analyze properties of a molecule based on intermolecular forces

U1: Propose a solution to a problem involving ideal gases or intermolecular forces providing appropriate justification of any choices made

S4 Course Grade Structure

A student's course grade reflects their demonstrated level of proficiency in Marzano's taxonomy. To effect this, the assessments account for 50 % of the course grade divided equally among the thirteen Knowledge Focuses. The student's grade on each assessment is the sum of the number of proficient responses. Student work demonstrating one proficiency is scored $1/4 = 25\,\%$; two proficiencies are scored $2/4 = 50\,\%$; etc. Of the three attempts, only the best score counts.

The remaining 50 % of the course score is divided among the laboratory (20 %), ALEKS homework (20 %), in-class participation (5 %), and ACS General Chemistry 1 paired question exam (5 %).

The course score percentages used to assign grades are summarized in Table S4. No

Table S4: Percentage of total course score to earn particular grades.

Mastery-based grading Select Anoth

**Name:**	**Range:**	
A	100 %	to 83.0%
A-	< 83.0 %	to 79.0%
B+	< 79.0 %	to 76.0%
B	< 76.0 %	to 69.0%
B-	< 69.0 %	to 67.0%
C+	< 67.0 %	to 63.0%
C	< 63.0 %	to 57.0%
C-	< 57.0 %	to 55.0%
D+	< 55.0 %	to 53.0%
D	< 53.0 %	to 47.0%
D-	< 47.0 %	to 45.0%
F	< 45.0 %	to 0%

"curve" or post-hoc adjustment to scores is applied. Given typical student averages on the homework, laboratory, and in-class participation, a student who scores > 3 in their assessments will obtain a course grade of A; > 2 a course grade of B; > 1 a course grade of C.

This approach is implemented in the Canvas LMS. Each Knowledge Focus is an "assign-

ment group" with a rule to drop the appropriate number of lowest scores (keep only the highest). Each Knowledge Focus is assigned 3.85 % of the course score. The other percentages are described above for the homework, laboratory, participation, and ACS exam assignment groups.

S5 Data Reduction Methods

To characterize the assessment items in the bank, we use Item Response Theory.[5,6] We calculate a one parameter logistic model (1PL, Rasch) to estimate of the ability of each student and the difficulty of each item.

The naive estimate of the difficulty of an assessment item i, β_i, is the negative logarithm of the odds ratio of a student answering proficiently

$$\beta_i = -\log\left(\frac{p_i}{1-p_i}\right),$$

where p_i is the probability of a correct response to that item, i.e., the number of correct attempts divided by the total number of attempts of the item. Higher, more positive values of β_i correspond to more difficult questions, and, conversely, lower, more negative values of β_i correspond to easier questions.

Item response theory also provides an estimate of a student's "ability". Item response theory is framed around ability as a latent trait of a student at a particular snapshot in time. As practitioners, we conceive of student ability as a quantity that can grow through student effort. Nevertheless, we adopt the term ability to match the technical language of the field.

With this caveat, student ability, θ_s, is estimated like item difficulty,

$$\theta_s = \log\left(\frac{p_s}{1-p_s}\right),$$

where p_s is the probability that a student will answer a question proficiently, i.e., the number

of proficient attempts divided by the total number of attempts for that student. Due to the structure of the class, not every student responds to each question or even attempts the same number of questions. As a result, different assessment items will have different numbers of total attempts, as will students. For the analysis of first attempts, the median number of responses is 40 (minimum 15, maximum 218).

The Rasch model proposes a logistic relationship between item difficulty and student ability. The probability that a student of ability θ_i correctly answers a question of difficulty β_j is

$$P(x_{ij} = 1|\theta_i, \beta_j) = \frac{1}{1 + \exp(-(\theta_i - \beta_j))}. \tag{S1}$$

where $x_{ij} = 1$ indicates a correct response. We used variational Bayesian inference to estimate the best fit item difficulties and student abilities as implemented in the Python package `py-irt`[7–9] with default settings. This package is particularly well suited because it handles sparse data (not every item has a response from every respondent).

To test for problematic assessment items, we calculate the point-biserial correlation[6] for each item,

$$r_i = \frac{M_{1,i} - M_{0,i}}{\sigma} \sqrt{p_i q_i} \tag{S2}$$

where i is the item, $M_{1,i}$ and $M_{0,i}$ are respectively the mean of the best scores of all assessments (on a scale of 0 to 4) of students who answered an item correctly or incorrectly, σ is the standard deviation of mean best scores, and p_i and q_i are the proportion of students who answered that particular question correctly or incorrectly. The point-biserial correlation gives a measure of the ability of a question to discriminate high- and low-performing students. When high-ability students tend to get a question correct and weak students tend to get the correction wrong, the r_i is high. If success on a question is weakly correlated with student ability, then the value of r_i is low. Standard definitions of item quality are poor ($r_i < 0.1$), fair ($0.1 \leq r_i < 0.2$), good ($0.2 \leq r_i < 0.3$), and very good ($r_i \geq 0.3$).[6]

S6 Item Response Theory of All Attempts

The structure of the IRT is very similar to the results considering only first attempts because very few students answer the same question more than once. The median number of responses per item is 66.5, with a range of 15 to 311.

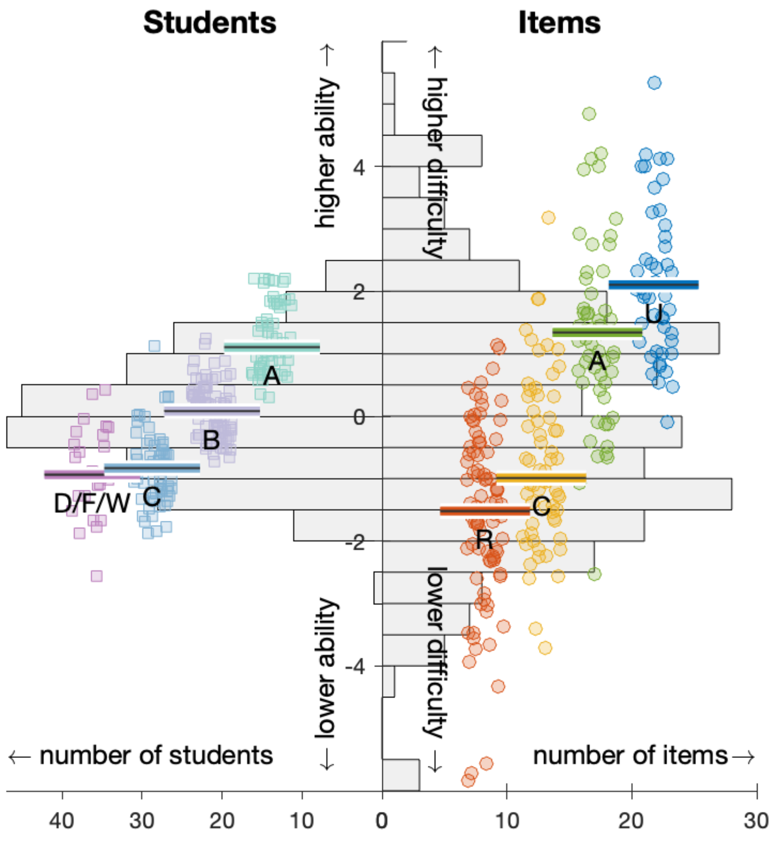

Figure S1: Item Response Theory using all attempts at assessment items provides the same structure as considering only first attempts.

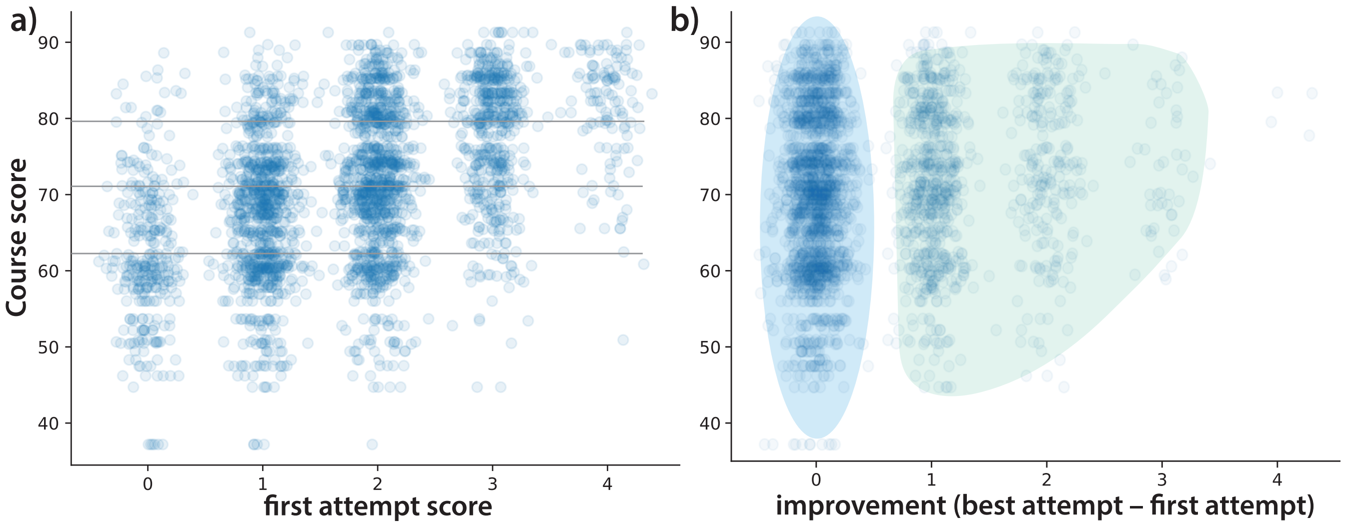

Figure S2: (a) Course score versus first attempt scores. Gray lines indicate the quartiles. Students in the lowest quartile typically score between 1 and 2 on their first attempt. Students in the hightest quartile typically score between 2 and 3 on their first attempt. (b) Course score versus improvement in assessment score. Students at all levels demonstrate improvement in assessment scores.

S7 Correlation of Course Score and Assessment Performance

The first attempt score has a low positive correlation with overall course score (Pearson correlation coefficient 0.47)(Figure S2a). Each students' score on each Knowledge Focus is represented as a point on the graphs (241 students, 13 Knowledge Focuses, ∼3000 data points). Each point reports the student's course score, on a 100 point scale, along the y-axis and (a) the initial score or (b) the score improvement on that Knowledge Focus, i.e., the assessment best attempt minus the first attempt, along the x-axis. The points are jittered along the x-axis for clarity. Students' first attempt scores are broadly distributed. Students with final course scores in the lowest quartile are most likely to score 1–2 and are very unlikely to score a 4 on a first attempt. Students in the highest quartile are most likely to score 2–3 and are very unlikely to score a 0 on a first attempt. Nevertheless, the distributions are very wide at all levels. This observation suggests that students come to the first attempt assessments with a broad range of skills.

Students across a wide range of course scores improve (Figure S2b). For each Knowledge Focus in which students did not improve, there is a point around an improvement (x-axis) of 0 (Figure S2, blue); 66 % of assessment attempts fall into this category. At the same time, 34 % of assessments show an increase of 1 to 3 points (Figure S2b, green). Students at all levels can improve, and the students who show the most improvement are among the top performers in the course overall.

S8 Minimal Working Example of the Codebase

The infrastructure used to create the assessments was based on open-source LaTeXpackages exam and pythontex, which are available through the Comprehensive TeXArchive Network, CTAN – `https://ctan.org`. CTAN provides ready-to-run TeXsystems for various platforms.

In the GitHub repository,[10] we have example code for selected questions from Knowledge Focus 1.1. These files serve as an minimal example of the code used to generate multiple versions of an assessment from a few general question stems.

1. Install LaTeX (`https://www.latex-project.org/get/`)
2. Install python > 3.9. (`https://www.anaconda.com/products/individual#Downloads`)
3. Install periodictable (`https://pypi.org/project/periodictable/`)
4. Install chempy (`https://pythonhosted.org/chempy/#installation`)

To test the installation, at a command prompt:

```
$  pdflatex -interaction=nonstopmode qtest.tex
$  pythontex --runall=true qtest.tex
$  pdflatex qtest.tex
$  pdflatex qtest.tex
```

To build five versions of the assessment:

```
$ python versions.py
```

References

(1) Marzano, R. J.; Kendall, J. S. *The New Taxonomy of Educational Objectives*, 2nd ed.; SAGE Publications, 2006.

(2) Webb, N. L. *Depth-of-knowledge levels for four content areas*; 2002; pp 1–9.

(3) Holme, T. A.; Luxford, C. J.; Brandriet, A. Defining Conceptual Understanding in General Chemistry. *Journal of Chemical Education* **2015**, *92*, 1477–1483.

(4) Ralph, V. R.; Scharlott, L. J.; Schafer, A. G.; Deshaye, M. Y.; Becker, N. M.; Stowe, R. L. Advancing Equity in STEM: The Impact Assessment Design Has on Who Succeeds in Undergraduate Introductory Chemistry. *JACS Au* **2022**, *2*, 1869–1880.

(5) Schurmeier, K. D.; Atwood, C. H.; Shepler, C. G.; Lautenschlager, G. J. Using Item Response Theory to Assess Changes in Student Performance Based on Changes in Question Wording. *Journal of Chemical Education* **2010**, *87*, 1268–1272.

(6) Sorenson, B.; Hanson, K. Using Classical Test Theory and Rasch Modeling to Improve General Chemistry Exams on a per Instructor Basis. *Journal of Chemical Education* **2021**, *98*, 1529–1538.

(7) Natesan, P.; Nandakumar, R.; Minka, T.; Rubright, J. D. Bayesian Prior Choice in IRT Estimation Using MCMC and Variational Bayes. *Frontiers in Psychology* **2016**, *7*.

(8) Lalor, J. P.; Wu, H.; Yu, H. Learning Latent Parameters without Human Response Patterns: Item Response Theory with Artificial Crowds. Proceedings of the 2019 Conference on Empirical Methods in Natural Language Processing and the 9th International Joint Conference on Natural Language Processing (EMNLP-IJCNLP). Stroudsburg, PA, USA, 2019; pp 4248–4258.

(9) Lalor, J. P.; Rodriguez, P. py-irt: A Scalable Item Response Theory Library for Python. **2022**,

(10) Shepherd, T. D.; Garrett-Roe, S. gotgchem-mwe. 2023; `https://github.com/sgarrettroe/gotgchem-mwe`.
